# Candidate Interventions for Integrating Hypertension and Cardiovascular-Kidney-Metabolic Care in Primary Health Settings: HEARTS 2.0 Phase 1

**DOI:** 10.5334/gh.1428

**Published:** 2025-05-27

**Authors:** Andres Rosende, Cesar Romero, Donald J. DiPette, Jeffrey Brettler, Patrick Van der Stuyft, Gautam Satheesh, Pablo Perel, Niamh Chapman, Andrew E. Moran, Aletta E. Schutte, James E. Sharman, Vilma Irazola, Mark D. Huffman, Norm R. C. Campbell, Abdul Salam, Fernando Lanas, Antonio Coca, Sebastian Garcia-Zamora, Alejandro Ferreiro, Patricio Lopez-Jaramillo, Jorge Rico-Fontalvo, Emily Ridley, Dean Picone, David Flood, Daniel José Piñeiro, Carolina Neira Ojeda, Gonzalo Rodriguez, Irmgardt A. Wellmann, Marcelo Orias, Marcela Rivera, Matías Villatoro Reyes, Oyere Onuma, Shaun Ramroop, Taskeen Khan, Yamile Valdes Gonzalez, Weimar Kunz Sebba Barroso, Frida L. Plavnik, Eric Zuniga, Ana María Grassani, Carlos Tajer, Ezequiel Zaidel, Marcos J. Marin, Shana Cyr-Philbert, Ignacio Amorin, Miguel Angel Diaz Aguilera, Luiz Bortolotto, Alvaro Avezum, Antonio Luiz P. Ribeiro, Sheldon Tobe, Teresa Aumala, Sonia Angell, Pablo Lavados, Sheila Ouriques Martins, Ana Munera Echeverri, Marc G. Jaffe, Dorairaj Prabhakaran, Gianfranco Parati, Xin Hua Zhang, Anthony Rodgers, Salim Yusuf, Paul K. Whelton, Pedro Ordunez

**Affiliations:** 1 Department of Noncommunicable Diseases and Mental Health, Pan American Health Organization, Washington, District of Columbia, USA; 2 Emory University, Atlanta, USA; 3 University of South Carolina, USA; 4 Kaiser Permanente, California, USA; 5 Department of Public Health and Primary care, Faculty of Medicine and Health Sciences, Ghent University, Gent, Belgium; 6 The George Institute for Global Health, Australia; 7 London School of Hygiene & Tropical Medicine, United Kingdom; 8 University of Sydney, Australia; 9 Columbia University Medical Center, USA; 10 Resolve to Save Lives, USA; 11 School of Population Health, University of New South Wales, Australia; 12 Menzies Institute for Medical Research, University of Tasmania, Hobart, Australia; 13 Department of Research in Chronic Diseases, Institute for Clinical Effectiveness and Health Policy (IECS), Buenos Aires, Argentina; 14 Washington University in St. Louis, USA; 15 The George Institute for Global Health, UNSW, Australia; 16 Department of Medicine, University of Calgary, Canada; 17 Universidad de La Frontera, Temuco, Chile; 18 Universitat Abat Oliba CEU, Barcelona, Spain; 19 Cardiology Department, Sanatorio Delta, Rosario, Argentina; 20 Centro de Nefrología, Facultad de Medicina, Universidad de la República, Uruguay; 21 Masira Research Institute, Medical School, Universidad de Santander (UDES), Bucaramanga, Colombia; 22 Departamento de Nefrología, Universidad Simón Bolivar, Barranquilla, Colombia; 23 Prisma Health, Columbia, South Carolina, USA; 24 School of Health Sciences, Faculty of Medicine and Health, University of Sydney, Australia; 25 Menzies Institute for Medical Research, University of Tasmania, Australia; 26 Department of Medicine, University of Michigan, USA; 27 World Heart Federation –University of Buenos Aires, Argentina; 28 Department of Noncommunicable Diseases, Ministry of Health of Chile, Santiago, Chile; 29 Hospital Universitario Fundación Favaloro, Buenos Aires, Argentina; 30 Research Center for Prevention of Chronic Diseases, Institute of Nutrition of Central America and Panama, Guatemala City, Guatemala; 31 Yale University School of Medicine, USA; 32 Division of Primary Healthcare, Ministry of Health of Chile, Santiago, Chile; 33 Oficina de Enfermedades No Transmisibles del Ministerio de Salud de El Salvador, El Salvador; 34 Harvard University School of Medicine, USA; 35 Chief Medical Officer of Bermuda, Bermuda; 36 Institute of Endocrinology, National Committee of Hypertension, Ministry of Public Health of Cuba, Cuba; 37 Hypertension Unit, Medicine School, Federal University of Goiás, Brazil; 38 Heart Institute of the Hospital das Clínicas of FMUSP –São Paulo, Brazil; 39 Universidad de Antofagasta, Servicio de Salud Antofagasta, Chile; 40 Ministry of Health of Tierra del Fuego, Ushuaia, Argentina; 41 Cardiology Department, Hospital El Cruce, Florencio Varela, Buenos Aires, Argentina; 42 Inter-American Society of Cardiology, USA; 43 Argentine Society of Hypertension, Argentina; 44 Ministry of Health, St. Lucia; 45 Universidad de la República, Montevideo, Uruguay; 46 National Center for Preventive Programs and Disease Control (CENAPRECE) of the Mexican Ministry of Health, Mexico; 47 InCor, Sao Paulo, Brazil; 48 Instituto de Cardiologia Dante Pazzanese, Sao Paulo, Brazil; 49 Department of Internal Medicine, Faculdade de Medicina, and Telehealth Center and Cardiology Service, Hospital das Clínicas, Universidade Federal de Minas Gerais, Belo Horizonte, Brazil; 50 University of Toronto and Northern Ontario School of Medicine, Toronto, Canada; 51 Instituto Ecuatoriano de Seguridad Social, Quito, Ecuador; 52 Johns Hopkins Bloomberg School of Public Health, USA; 53 Iberoamerican Stroke Society, Facultad de Medicina, Clinica Alemana, Universidad del Desarrollo, Santiago, Chile; 54 World Stroke Organization, Switzerland; 55 Centre for Chronic Disease Control, New Delhi, India; 56 IRCCS, Italian Auxology Institute, San Luca Hospital, Milan, Italy; 57 Department of Medicine and Surgery, University of Milan-Bicocca, Milan, Italy; 58 Beijing Hypertension League Institute, China; 59 McMaster University, Ontario, Canada; 60 World Hypertension League, Tulane University, New Orleans, Louisiana, USA

**Keywords:** hypertension, Primary Health Care, Cardiovascular Diseases, diabetes mellitus, Renal Insufficiency, Chronic, Stroke

## Abstract

**Background:**

HEARTS in the Americas is the regional adaptation of the WHO Global HEARTS Initiative, aimed at helping countries enhance hypertension and cardiovascular disease (CVD) risk management in primary care settings. Its core implementation tool, the HEARTS Clinical Pathway, has been adopted by 28 countries. To improve the care of hypertension, diabetes, and chronic kidney disease (CKD), HEARTS 2.0 was developed as a three-phase process to integrate evidence-based interventions into a unified care pathway, ensuring consistency across fragmented guidelines. This paper focuses on Phase 1, highlighting targeted interventions to improve and update the HEARTS Clinical Pathway.

**Methods:**

First, the coordinating group defined the project’s scope, objectives, principles, methodological framework, and tools. Second, international experts from different disciplines proposed interventions to enhance the HEARTS Clinical Pathway. Third, the coordinating group harmonized these proposals into unique interventions. Fourth, experts appraised the appropriateness of the proposed interventions on a 1-to-9 scale using the adapted RAND/UCLA Appropriateness Method. Finally, interventions with a median score above 6 were deemed appropriate and selected as candidates to enhance the HEARTS Clinical Pathway.

**Results:**

Building on the existing HEARTS Clinical Pathway, 45 unique interventions were selected, including community-based screening, early detection and management of risk factors, lower blood pressure thresholds for diagnosing hypertension in high-CVD-risk patients, reinforcement of single-pill combination therapy, inclusion of sodium-glucose cotransporter-2 inhibitors for patients with diabetes, CKD, or heart failure, expanded roles for non-physician health workers in team-based care, and strengthened clinical documentation, monitoring, and evaluation.

**Conclusion:**

HEARTS 2.0 Phase 1 identifies key interventions to integrate and improve hypertension and cardiovascular-kidney-metabolic care within primary care, enabling their seamless incorporation into a unified and effective clinical pathway. This process will inform an update to the HEARTS Clinical Pathway, optimizing resources, reducing care fragmentation, improving care delivery, and advancing health equity, thereby supporting global efforts to combat the leading causes of death and disability.

## Introduction

The World Health Organization’s (WHO) Global HEARTS Initiative (1) supports health ministries in developing cardiovascular disease (CVD) prevention strategies, focusing on hypertension management. HEARTS in the Americas, the regional adaptation coordinated by the Pan American Health Organization (PAHO), currently reaches 37 million adults, with 5.7 million receiving hypertension treatment, across over 7,200 primary healthcare (PHC) centers in 33 countries. The program aims to transform health services and clinical practice to enhance hypertension control and integrated CVD risk management in PHC (2). High systolic blood pressure (SBP) is the main risk factor for ischemic heart disease (IHD) and stroke (3), and countries with the highest levels of population hypertension control tend to have low IHD and stroke mortality rates (4).

The HEARTS Clinical Pathway, the central tool for HEARTS in the Americas (5), aligns with the 2021 WHO pharmacologic hypertension guidelines (6) and is shaped by HEARTS control drivers (7). It features a standardized treatment protocol, initiating at blood pressure (BP) ≥140/90 mmHg, with monthly intensifications until BP is controlled (8), prioritizing long-acting agents and single-pill antihypertensive combinations (SPC) (9). CVD risk is assessed using WHO risk charts, with high risk defined by IHD, stroke, diabetes mellitus, CKD, or a 10-year CVD risk ≥10%. Immediate treatment is recommended for high-risk patients with SBP ≥130 mmHg, with rapid titration to reach a SBP <130 mmHg. High-intensity statins and low-dose aspirin are advised for patients with established CVD, while moderate-intensity statins without aspirin are recommended for those patients at high risk but without established CVD (10).

The HEARTS Clinical Pathway, currently implemented in 28 countries across Latin America and the Caribbean region, has been essential in standardizing management in implementing countries (11). This tool also commits health authorities to ensure access to high-quality, affordable essential medicines, clinically validated automated blood pressure measurement devices (BPMD), and other critical resources (12). Designed for broad implementation at the PHC level, it promotes a team-based care model that involves patients and non-physician health workers (NPHW).

The rising prevalence of obesity, hypertension, and diabetes, coupled with the emergence of the Cardiovascular-Kidney-Metabolic Syndrome (CKM)—a serious health disorder attributable to connections among obesity, diabetes, CVD, and CKD—underscores the urgent need to adopt innovative clinical practices (13,14). Additionally, HEARTS countries have called for more comprehensive management tools for noncommunicable diseases (NCDs), while PAHO’s Better Care for NCDs advocates for an integrated approach to strengthening PHC capacity (15). These factors highlight the pressing need to update the current HEARTS Clinical Pathway.

To advance the HEARTS Clinical Pathway for cardio-renal-metabolic management, we developed HEARTS 2.0—a three-phase process to integrate and align evidence-based interventions into a unified care pathway. Phase 1 identifies synergistic interventions for hypertension, CVD prevention, diabetes mellitus, and CKD that can be integrated into the pathway. Phase 2 will assess countries’ readiness for implementation, while Phase 3 will select interventions to update the pathway and develop strategies for broader adoption. This stepwise approach ensures participation from countries and stakeholders, reinforcing the inclusive and evidence-based framework of HEARTS in the Americas.

This paper describes the design, conduct, and outcomes of Phase 1 of HEARTS 2.0, presenting a detailed list of candidate interventions for integration into the updated HEARTS Clinical Pathway. The aim is to create a more cohesive and integrated pathway that enhances multimorbidity management, reduces care fragmentation, and fosters coordinated action within primary healthcare systems.

## Methods

HEARTS 2.0 Phase 1 followed a systematic and multi-step process. First, the coordinating group defined the project’s scope, objectives, and overarching principles, and created data collection tools. Second, a group of multidisciplinary international subject-matter experts, familiar with both the Global HEARTS initiative and HEARTS in the Americas was invited to propose interventions to enhance the current HEARTS Clinical Pathway. Third, the coordinating group reviewed and consolidated the improvement proposals, leading to the identification of unique interventions. Fourth, experts appraised the appropriateness of the proposed interventions for relevance and suitability. Fifth, the highest-scoring interventions were deemed appropriate and selected as candidates to enhance the HEARTS Clinical Pathway.

**Figure 1:**
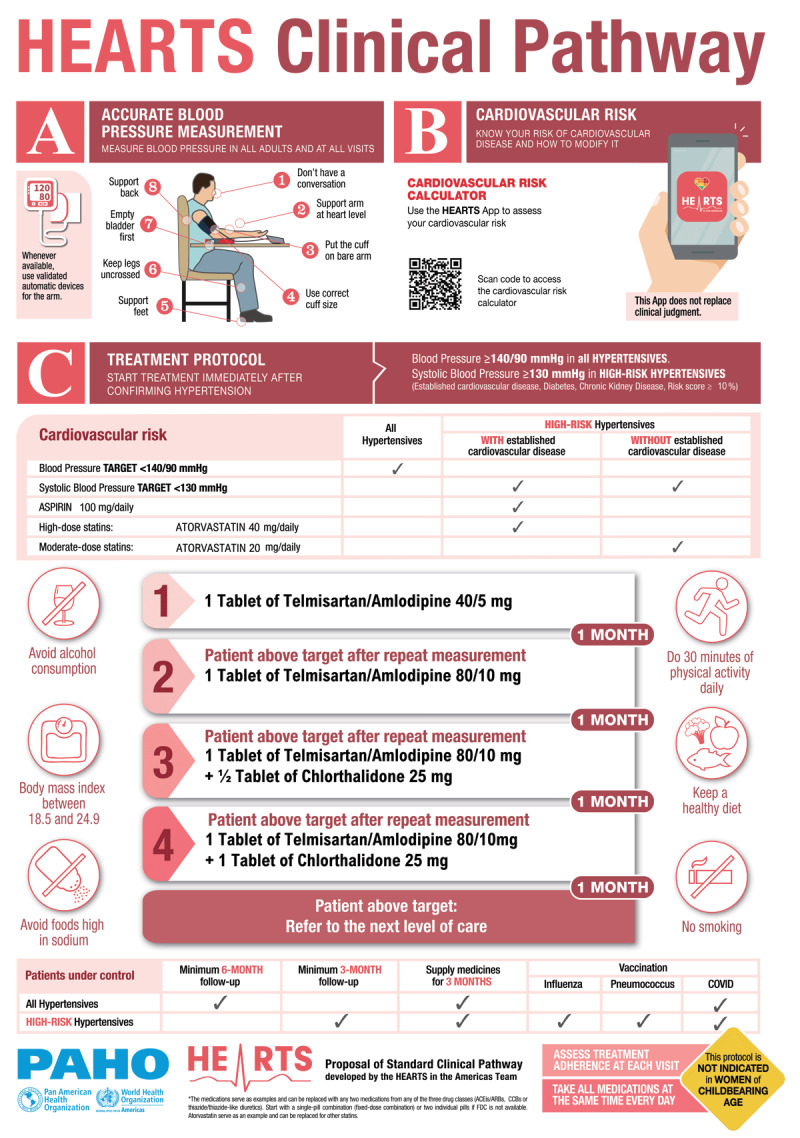
The HEARTS Clinical Pathway.

The project adhered to three overarching principles: first, all interventions should build upon the existing HEARTS Clinical Pathway (Figure 1); second, new interventions (whether modifications or additions) would only be considered if supported by robust scientific evidence, prioritizing those endorsed by leading clinical practice guidelines or widely recognized as beneficial through international consensus; and third, each intervention had to be feasible and safe for large-scale implementation in diverse PHC settings.

Seventy-one international experts were invited reflecting diverse representation across geographical regions, income levels, demographics, professional backgrounds, and research experience, including key representatives from global and regional organizations developing relevant clinical guidelines. Experts were asked to propose up to three impactful interventions in areas covered by the HEARTS Clinical Pathway, such as diagnosis, risk assessment, treatment, continuity of care, delivery systems, immunization, and follow-up. For each proposal, experts were instructed to propose only interventions supported by robust evidence, particularly those endorsed by leading guidelines or international consensus. They also provided references for supporting evidence and a justification for each intervention.

The coordinating group reviewed and consolidated the proposed interventions, which were then presented to the experts, along with their intervention areas, recommended actions (remove, reinforce, modify, or include), and supporting evidence (see “Appropriateness Exercise Instrument” in the supplementary materials). Experts rated each intervention for appropriateness using the RAND/UCLA Appropriateness Method (16). This method consists in a systematic approach to evaluate the suitability of specific interventions, tests, or procedures for clinical scenarios or populations. These assessments combine expert judgment, clinical guidelines, and scientific evidence to ensure that interventions align with best practices and are contextually relevant. Experts rate the appropriateness of interventions using a scale of 1 to 9, and appropriateness is defined based on the expected health benefit outweighing the expected negative consequences. Each intervention is categorized as appropriate if the median of the ratings falls in the top third, uncertain if the median falls in the middle third, and inappropriate if the median of the responses falls in the bottom third. Since experts were free to respond according to their expertise, and to avoid bias, the medians were weighed by the response rate.

Finally, the assessment results were shared with all experts for additional comments or objections. Interventions with a median score above 6 were deemed appropriate and selected as candidates to enhance the HEARTS Clinical Pathway.

## Results

**Figure 2:**
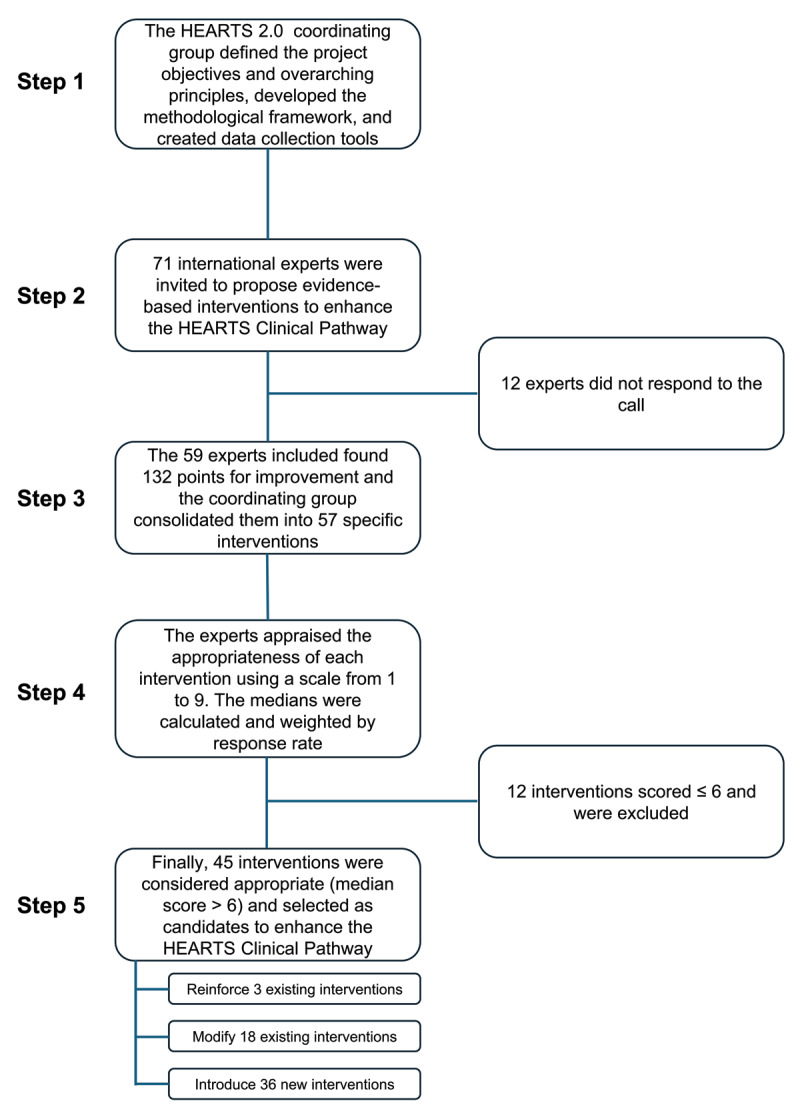
Flowchart summarizing the process for evaluating the appropriateness of candidate interventions to be included in the HEARTS Clinical Pathway.

Figure 2 summarizes the process for evaluating the appropriateness of candidate interventions to be incorporated into the HEARTS Clinical Pathway. Of the 71 experts invited, 59 responded to the call (see Table “Characteristics of experts” in supplementary materials), contributing 132 proposals for improvement. These proposals were consolidated into 57 specific interventions, highlighting the acceptability of the current HEARTS Clinical Pathway—no interventions were recommended for removal—and multiple opportunities for enhancement.

The assessment resulted in three key actions for improvement: a) reinforcing three existing interventions of significant value that could benefit from greater emphasis; b) modifying 18 existing components to align with current best practices; and c) introducing 36 new specific interventions to address emerging challenges and incorporate recent advancements into the pathway (Table 1).

**Table 1:** Number of improvement interventions agreed by action and area.


ACTION RECOMMENDED	DIAGNOSIS	RISK ASSESSMENT	NON-PHARMACOLOGIC TREATMENT	PHARMACOLOGIC TREATMENT	CONTINUITY OF CARE	DELIVERY SYSTEM	SYSTEM FOR MONITORING	IMMUNIZATION	TOTAL

**Include**	3	8	7	6	7	3	2	0	**36**

**Modify**	3	4	0	6	2	0	0	3	**18**

**Reinforce**	2	0	0	1	0	0	0	0	**3**

**Remove**	0	0	0	0	0	0	0	0	**0**

**With high median score**	**4/8**	**9/12**	**6/7**	**13/13**	**6/9**	**3/3**	**2/2**	**2/3**	**45/57**


A total of 78.9% (45 out of 57) of the proposed interventions received high appropriateness scores and a strong expert consensus on the value of these changes. These high-rated interventions, summarized in Table 2, encompass all areas of the HEARTS Clinical Pathway, signaling a comprehensive strategy to enhance cardiovascular care. The results of this appraisal can be summarized as follows:

**Table 2:** Candidate Interventions for the HEARTS Clinical Pathway Upgrade.


INTERVENTION AREAS	ACTION TO BE TAKEN	IMPROVEMENT PROPOSALS AGREED

**Diagnosis**	**Reinforce**	**1.** Exclusive use of clinically validated BPMD.

		**2.** Improving the clinical environment for accurate BP measurement.

	**Include**	**3.** Expanding community outreach for hypertension screening.

		**4.** Set BP diagnostic thresholds for hypertension at ≥140/90 mmHg in the general population and SBP ≥130 mmHg for patients at high cardiovascular risk.

**Risk assessment**	**Modify**	**5.** Set CKD definition: eGFR < 60 ml/min and/or uACR index ≥ 30 mg/g.

		**6.** Set BP goals in elderly patients to SBP <130 mmHg.

		**7.** Define a CVD risk approach for young adults (18–40 years).

	**Include**	**8.** CKD screening for high-risk individuals using uACR and eGFR.

		**9.** Assessment of HTN-mediated organ damage with ECG in high CVD risk patients.

		**10.** Screening for dyslipidemia and diabetes in patients with HTN and obesity.

		**11.** Opportunistic screening for atrial fibrillation in high CVD risk patients of any age and those aged ≥ 65 years.

		**12.** Closely monitor individuals with a history of hypertension during pregnancy.

		**13.** Add a warning: Avoid treatment with short-acting and parenteral agents in individuals with severe asymptomatic uncontrolled hypertension.

**Non-Pharmacologic Treatment**	**Include**	**14.** Promote low-sodium /potassium-enriched salt.

		**15.** Prescribe isometric exercise.

		**16.** Warning against smoking Cannabis.

		**17.** Warning against Electronic Cigarette use/Vaping.

		**18.** Avoid sedentary lifestyle.

		**19.** Prescribe exercise.

**Pharmacologic Treatment**	**Reinforce**	**20.** SPC antihypertensive medicines.

	**Modify**	**21.** Add the third drug, at half maximum dose, in the second step of the treatment protocol instead of increasing the first two drugs to maximum doses.

		**22.** Maximum statin doses in secondary prevention (Atorvastatin 80 mg or Rosuvastatin 40 mg).

		**23.** High statin doses in primary prevention (Atorvastatin 40 mg or Rosuvastatin 20 mg).

		**24.** Use of Polypills (antihypertensives plus statins with or without aspirin) for primary and secondary prevention of CVD.

		**25.** Intensify antihypertensive medication at intervals of 2 weeks instead of 4 weeks.

		**26.** Warning on assessing childbearing potential before treatment initiation.

	**Include**	**27.** Triple SPC for those patients who do not reach BP control using double SPC.

		**28.** Spironolactone in patients with 3 drugs at maximum doses and lack of HTN control.

		**29.** Treatment for tobacco cessation (bupropion, varenicline, nicotine substitutes).

		**30.** Prescribe SGLT2i in patients with CKD.

		**31.** Prescribe SGLT2i in patients with heart failure.

		**32.** Prescribe SGLT2i in patients with diabetes and established CVD.

**Continuity of Care**	**Modify**	**33.** Intensive BP (SBP < 130 mmHg) goals restricted to patients <80 years.

	**Include**	**34.** Home BP treatment monitoring.

		**35.** Telemedicine/mHealth apps to monitor recommendation adherence and to reduce loss to follow-up.

		**36.** Lipid targets in high CVD risk patients.

		**37.** An established target time to achieve BP control.

		**38.** Warning to avoid statin discontinuation once the control target has been reached.

**Delivery System**	**Include**	**39.** Medication intensification by non-physician healthcare workers following a protocol.

		**40.** HTN screening and CVD risk stratification by non-physician healthcare workers.

		**41.** Healthy lifestyle counseling and support for medication adherence by non-physician healthcare workers.

**Immunization**	**Modify**	**42.** Influenza vaccination to all patients with HTN regardless of CVD risk level.

		**43.** Pneumococcus vaccination should exclude patients in primary prevention <65 years.

**System for Monitoring**	**Include**	**44.** Importance of registering clinical variables.

		**45.** Relevance of having a strategy of performance evaluation with feedback.


BP: blood pressure; BPMD: blood pressure measuring devices; HTN: hypertension; CVD: cardiovascular disease; CKD: chronic kidney disease; eGFR: estimated glomerular filtration rate; AlbU: urine albumin; CrU: urine creatine; uACR: urine albumin-creatinine ratio; ECG: electrocardiogram; AF: atrial fibrillation; SPC: single pill combination; SGLT2i: sodium-glucose co-tranporter-2 inhibitors.

Many proposed interventions focused on early detection and intensive management of risk factors like hypertension, dyslipidemia, diabetes, CKD and atrial fibrillation (AF), underlining a preventative approach to cardiovascular health.

The interventions emphasized integrating lifestyle modifications such as a healthy diet, reduced-sodium/potassium-enriched salt consumption, and increased physical activity, while also highlighting the risks of cannabis and electronic cigarette use and reinforcing the general recommendation against tobacco use, to promote a holistic approach to patient management.

The experts stressed the expanded use of SPCs to simplify medication regimens and improve treatment adherence. They also recommended adjusting statin doses for CVD primary and secondary prevention, with maximum doses specifically suggested for those with existing CVD. Furthermore, the experts highlighted the potential benefits of sodium-glucose cotransporter-2 inhibitors (SGLT2i) for patients with diabetes, CKD, or heart failure.

Expanding the role of NPHWs was seen as crucial to improve access to care, provide patient education and support, and potentially handle tasks like medication titration under supervision, suggesting a more team-based approach to care delivery.

The experts emphasized the importance of targeted vaccination strategies to improve patient outcomes. They supported universal COVID-19 vaccination and recommended expanding influenza vaccination to include all individuals with hypertension. For pneumococcal vaccination, the experts advised prioritizing individuals aged 65 and older, focusing on those at higher risk.

Finally, the experts underscored the importance of a robust monitoring and evaluation system. They advocated systematic documentation of clinical variables, such as BP values and CVD risk levels, and recommended incorporating a performance evaluation strategy with feedback into the HEARTS Clinical Pathway.

The detailed breakdown of all 57 interventions, including supporting evidence and expert ratings, is available in the supplementary materials (“Results of the Appropriateness Exercise” and “Agreed Interventions and Their Supports” tables).

## Discussion

HEARTS 2.0 was designed to integrate cardiovascular-kidney-metabolic care into primary care and foster synergy across multiple evidence-based interventions. Building on a robust clinical pathway already adopted by most countries in the Americas (4,5,11,17), this framework aims to address the complex needs of patients with multiple risk factors and conditions by prioritizing clinically proven solutions while emphasizing public health and health system strategies. Hypertension management remains the cornerstone of the HEARTS Clinical Pathway, anchoring other critical preventive interventions, while the PHC setting provides the foundation for delivering coordinated, people-centered care (4,18). As far as we know, this is the first initiative of its kind and scope globally.

Strengthening interventions for accurate BP measurement aligns with global efforts to standardize BP measurement in clinical settings (19). This underscores the need for stricter regulations mandating the validation of automatic BPMDs (20) because non-validated devices increase the risk of inaccurate readings, misdiagnosis, and mistreatment of hypertension (21). Misconceptions around market approval requirements have led to the proliferation of non-validated BPMDs, with fewer than 20% of automated upper-arm and wrist devices meeting internationally accepted validation standards (22). HEARTS in the Americas promotes BP measurement accuracy including a regulatory framework mandating clinically validated BPMDs in PHC (23).

The current HEARTS Clinical Pathway already promotes a differentiated approach for people with diabetes or CKD, recommending more intensive hypertension management to achieve a target SBP of <130 mmHg (24), along with the use of statins or aspirin (10). However, it does not include guidance on how to identify these patients. For that reason, a key improvement focuses on opportunistic screening for CKD in high-risk individuals, using urine albumin-creatinine ratio (uACR) and estimated glomerular filtration rate (eGFR) thresholds (25). Hypertension remains prevalent and poorly controlled (26), while type 2 diabetes mellitus continues to rise without sufficient expansion in treatment (27,28). Together, these conditions drive over 75% of CKD risk, worsening both hypertension and diabetes and elevating CVD risk and premature death (14). Indeed, nearly 90% of those with CKM Syndrome die from CVD before reaching end-stage kidney disease (14). Intensifying BP control to SBP <130 mmHg in high-CVD risk patients, including those with diabetes or CKD, is recommended by 2021 WHO hypertension guideline (6) and is one of the most effective interventions to reduce CVD mortality (29) and slow CKD progression (30).

Hypertension, obesity, and aging are risk factors for AF (31,32). Opportunistic AF screening for high-CVD-risk patients could enhance the HEARTS Clinical Pathway, in line with evidence (33) and clinical guidelines (34). AF affects 10% of people aged 65 and older, with its global burden doubling since 1990 (35). AF increases stroke risk fivefold, often leading to disabling or fatal strokes. Early detection and anticoagulation can reduce stroke risk by 70%; yet 30% of individuals remain undiagnosed (34). Integrating simple, cost-effective AF case-finding into the HEARTS pathway could improve detection and access to anticoagulants, enhancing clinical outcomes (36).

Replacing regular salt with reduced-sodium/potassium-enriched salt significantly lowers BP, reduces CVD incidence, and decreases all-cause mortality (37). Adding this cost-effective intervention to the HEARTS Clinical Pathway could reduce BP and CVD risk for millions of adults in the HEARTS in the Americas program (38). Despite poor implementation, it has strong global support as an impactful measure (39,40,41).

Experts agreed on strengthening physical activity recommendations by advising patients on sedentary behavior and prescribing increased physical activity, supported by evidence (42,43) and clinical guidelines (44,45). They also recommended adding warnings about e-cigarettes/vaping, and cannabis use due to their growing use and link to CVD (46,47). These non-pharmacologic strategies reinforce the HEARTS’ role in empowering communities and engaging patients in their care.

The experts strongly supported expanding SPC use to address treatment inertia, medication adherence, persistence, and effectiveness of hypertension programs, including introducing triple SPC for patients who do not achieve BP control with double SPC (48). SPCs are recommended by all major guidelines, (6,49,50,51) and were included in the WHO essential medicines list (52), but adoption is hindered by procurement cost, physician reluctance, and restricted access (53). Barriers include outdated national essential medicines lists, limited market availability, fragmented procurement processes, and exclusion from insurance programs or public sector provision, leading to high out-of-pocket costs. Leveraging pooled procurement mechanisms, like the PAHO Strategic Fund, and updating national essential medicines lists could improve accessibility and affordability (54).

The availability of SGLT2i has shifted the focus of diabetes care beyond glucose control to include reducing CVD risk and preventing CKD progression (25,55). Together with the HEARTS Clinical Pathway’s recommendations for more stringent hypertension targets in patients with diabetes and CKD (5) and higher statin doses (56), these interventions form a comprehensive approach for patients with multiple conditions including the CKM syndrome. This strategy is primed for rapid expansion, as the WHO has included SGLT2i in its essential medicines list (52) and PAHO has added them to the Strategic Fund’s procurement list (57).

A key improvement intervention was adopting a structured, time-sensitive approach to hypertension control. This includes shorter medication titration intervals, earlier treatment intensification, and time-bound BP targets to reduce CVD risk (58). Acknowledging that delays in BP control increase adverse outcomes, the pathway promotes timely interventions and follow-up visits within three months for high CVD-risk patients and six months for others, fostering sustained BP control, further improving CVD outcomes.

The experts supported HBPM and telemedicine/mHealth as key improvements. While HBPM is recommended by major clinical guidelines (49,50,51), its implementation is limited by access to clinically validated BPMDs. Telemedicine offers a potential solution for improving access to care, continuity, and follow-up, especially in underserved areas, crucial for maintaining BP control and preventing complications (59).

The experts supported reinforcing NPHW’s role in hypertension screening, patient education, and treatment intensification using the HEARTS Clinical Pathway. A recent trial showed that NPHW-led intervention improved hypertension control and reduced CVD and mortality (60). However, regulatory and cultural barriers in many HEARTS-implementing countries hinder broader implementation (5,61,62).

The current HEARTS Clinical Pathway recommends influenza vaccination for high CVD-risk patients. However, the experts suggested this recommendation to all individuals with hypertension, regardless of their CVD risk, based on evidence that infectious diseases can act as triggers for cardiovascular events, while immunization reduces their occurrence. Influenza vaccination is highly cost-effective, supporting its widespread adoption (63).

### Strengths and limitations

HEARTS 2.0 Phase 1 is not designed to create clinical guidelines or position statements. Instead, it follows a systematic and rigorous appropriateness assessment process. Led by a group of multidisciplinary international experts, it aims to integrate synergistic, evidence-based interventions from existing guidelines to streamline the HEARTS Clinical Pathway and address related conditions in PHC settings. It reduces costly evaluations by harmonizing interventions already endorsed by various authorities. Indeed, key interventions identified by the experts are already part of major clinical guidelines and position statements. However, a limitation is that interventions were not assessed in terms of health budget impact or cost-effectiveness; as country-level cost and cost-effectiveness assessments were beyond the scope of the HEARTS 2.0 process. Recognizing that evidence from clinical trials may not always apply to real-world practice, further research is needed for broad implementation.

Although HEARTS promotes teamwork, and three interventions on this topic were considered appropriate, a limitation of the expert group is that almost 90% of its members are medical doctors. However, this distribution reflects the broader overrepresentation of physicians in the research field as well as in the development of clinical practice guidelines.

Finally, all experts approved the methodology and results and submitted conflict-of-interest declarations. Expert anonymity was maintained to prevent cross influence, and results were shared for feedback. Any experts involved in Phase 1 who declared any conflict of interest during this process will be excluded from HEARTS 2.0 Phase 3, which will focus on selecting interventions to update the clinical pathway and developing strategies for broader adoption.

### Key findings and next steps

The experts acknowledged the high technical quality of the current HEARTS Clinical Pathway while identified several areas for improvement. Proposed innovations included community-based screening, early detection and management of risk factors, guideline-aligned BP thresholds for diagnosing hypertension in high-CVD risk patients, reinforcement of the SPC recommendation, the inclusion of new treatments for diabetes and CKD, enhanced clinical monitoring of hypertension, expanded roles of NPHWs in team-based care, and strengthening clinical documentation, monitoring and evaluation.

In HEARTS 2.0 Phase 1, experts identified candidate interventions to improve the HEARTS Clinical Pathway. As implementation approaches, the dialogue will expand to include policymakers, healthcare workers, patients, and other stakeholders in HEARTS-implementing countries. The second phase of HEARTS 2.0 will map countries’ preparedness and readiness to implement these interventions, guiding future decision-making. In the third phase, a panel will prioritize interventions for inclusion in the updated clinical pathway. Some interventions may require further evaluation, while those with strong evidence but low feasibility for implementation will be advanced through advocacy and policy efforts.

## Conclusion

The improvement interventions identified in HEARTS 2.0 Phase 1, grounded in evidence-based principles and multidisciplinary collaboration, have the potential to transform CVD management in PHC settings. This initiative is crucial given the persistent prevalence of hypertension, the rise in type 2 diabetes, and their link to CKD. The development of an updated HEARTS Clinical Pathway will be key to optimizing resources, reducing care fragmentation, improving care delivery, and enhancing equity. Ultimately, this will push the boundaries of PHC to more effectively address the leading causes of death and disability worldwide.

## Disclaimer

The authors are staff members of the Pan American Health Organization. The authors alone are responsible for the views expressed in this publication and they do not necessarily represent the views, decisions or policies of the Pan American Health Organization.

## Additional File

The additional file for this article can be found as follows:

10.5334/gh.1428.s1 Supplementary materials. HEARTS 2.0 Phase 1. This file contains information on the composition of the group of experts who participated in the consultation, as well as the instrument used to select the candidate interventions to integrate the new clinical pathway and their support in evidence.
