# Supplementary material for: Candidate Interventions for Integrating Hypertension and Cardiovascular-Kidney-Metabolic Care in Primary Health Settings: HEARTS 2.0 Phase 1
Source: Glob Heart. 2025 May 27;20(1):45. doi: 10.5334/gh.1428 (PMC12124280; doi:10.5334/gh.1428)
Supplement: Supplementary materials. — HEARTS 2.0 Phase 1. This file contains information on the composition of the group of experts who participated in the consultation, as well as the instrument used to select the candidate interventions to integrate the new clinical pathway and their support in evidence. [file gh-20-1-1428-s1.pdf]

Characteristics of experts included

Main characteristics of the expert group	
**Sex**	
Female	18 (30.5%)
Male	41 (69.5%)
**Region**	
America	45 (76.3%)
North America	15 (33.3%)
Central America & the Caribbean	5 (11.1%)
South America	25 (55.6%)
Other*	14 (23.7%)
**Profession**	
Medical doctor	53 (89.8%)
Other**	6 (10.2%)
**Main Specialty**	
Cardiology	18 (30.5%)
Hypertension	7 (11.9%)
Public Health	11 (18.6%)
Other***	23 (39%)
**Academic profile**	
H-index (median)	27.5
H-index≥20 (n;%)	29 (53,7%)

*Includes experts from Asia, Europe, and Oceania

**Includes nurses, pharmacists, researchers, and university professors.

***Includes Endocrinology, Epidemiology, Family Medicine, Internal Medicine, Neprhology, and Neurology

N°	Intervention Area	Action	Improvement Proposal
1	Diagnosis	Reinforce	Exclusive use of clinically validated Blood Pressure Measuring Devices to accurate BP measurement
2	Diagnosis	Include	A recommendation for BP mesurement in patients with atrial fibrillation.
3	Diagnosis	Modify	Simplify HTN diagnosis by using the 2nd BP measurement only when the 1st one is at least 130/80 mmHg.
4	Diagnosis	Modify	Simplify HTN diagnosis by using the 2nd BP measurement only when the 1st one is at least 140/90 mmHg. A 3rd measurement will be used if the difference between either the first two systolic or diastolic BPs was >5 mmHg.
5	Diagnosis	Modify	Avoid the 5 minutes of rest before BP measurement.

**6**	**Diagnosis**	**Reinforce**	Recommendations to improve BP measurement (unobserved, quiet room, etc).
**7**	**Diagnosis**	**Include**	Expand HTN screening in the community served by each PHC (community engagement).
**8**	**Diagnosis**	**Include**	BP thresholds to consider HTN in the step A (BP 140/90 in general population and SBP 130 in high CVD risk).
**9**	**Risk assessment**	**Include**	Screening of CKD by urine albumin-creatinine ratio (uACR) and estimated Glomerular Filtration Rate (eGFR).

**10**	**Risk assessment**	**Modify**	De-prioritizing the use of CVD risk charts in CVD risk assessment.
**11**	**Risk assessment**	**Modify**	Clarify the CKD definition as follows: eGFR < 60 ml/min and/or AlbU/CrU index ≥ 30 mg/g.
**12**	**Risk assessment**	**Include**	A case finding strategy (opportunistic screening) for Atrial Fibrillation, in high CVD risk patients of any age and in those ≥ 65 years, using a stepwise approach: radial pulse palpation to all and ECG in those with 1st test possitive.

**13**	**Risk assessment**	**Modify**	BP goals in elderly patients to SBP <130 (age ≥ 65 years as a high CVD-risk equivalent).
**14**	**Risk assessment**	**Include**	History of HTN during pregnancy in the CVD risk assessment.
**15**	**Risk assessment**	**Modify**	Clarify the CVD risk approach for young adults (18 - 40 years) who are

			not covered by the CVD risk charts.
**16**	**Risk assessment**	**Include**	A Recommendation to measure HTN-mediated organ damage with ECG in high CVD risk patients.
**17**	**Risk assessment**	**Include**	Measure the heart rate variability using a smartphone (photopletysmography)
**18**	**Risk assessment**	**Include**	Assess physcological distress using a simple tool such as Patient Health Questionnaire -2 Depression Screen.

**19**	**Risk assessment**	**Include**	Screening for dyslipidimia and diabetes ammong patients with hypertension and obesity.
**20**	**Risk assessment**	**Include**	Warning on the treatment of asymptomatic severe HTN to avoid referral to emergency department and acute treatment with short-acting/parenterally agents.
**21**	**Non-Pharmacologic treatment**	**Include**	A recommendation on the consumption of low-sodium / high-potassium salt.

**22**	**Non-Pharmacologic treatment**	**Include**	A recommendation on isometric exercise.
**23**	**Non-Pharmacologic treatment**	**Include**	A recommendation on sleep quality - Circadian Synchrony.
**24**	**Non-Pharmacologic treatment**	**Include**	Warning against Cannabis use.
**25**	**Non-Pharmacologic treatment**	**Include**	Warning against Electronic Cigarette use.
**26**	**Non-Pharmacologic treatment**	**Include**	A recommendation to avoid the sedentary lifestyle.

**27**	**Non-Pharmacologic treatment**	**Include**	A recommendation on excersice prescription.
**28**	**Pharmacologic treatment**	**Reinforce**	Use of fixed-dose combination.
**29**	**Pharmacologic treatment**	**Include**	Recommendation of Triple FDC for those patients who don´t reach BP control using Double FDC.
**30**	**Pharmacologic**	**Modify**	Add the third drug, at half maximum dose, in the second step of the treatment protocol instead increasing

**30**	**treatment**	**Modify**	treatment protocol instead increasing the first two drugs to maximum doses.
**31**	**Pharmacologic treatment**	**Include**	Spironolactone in patients with 3 drugs at maximum doses and lack of HTN control.
**32**	**Pharmacologic treatment**	**Modify**	Statin dose in secondary prevention to Atorvastatin 80 mg or Rosuvastatin 40 mg.
**33**	**Pharmacologic treatment**	**Modify**	Statin dose in primary prevention to Atorvastatin 40 mg or Rosuvastatin 20 mg.

**34**	**Pharmacologic treatment**	**Modify**	Replace current medications in the treatment protocol with polypills (antihypertensive + statin +/- aspirin) for primary and secondary prevention.
**35**	**Pharmacologic treatment**	**Include**	A recommendation for tobacco cessation treatment (e.g. bupropion, varenicline, nicotine substitutes)
**36**	**Pharmacologic treatment**	**Include**	Recommendation to use iSGLT2 in patients with CKD.

**37**	**Pharmacologic treatment**	**Include**	Recommendation to use iSGLT2 in patients with heart failure, regardless of ejection fraction.
**38**	**Pharmacologic treatment**	**Include**	Recommendation to use iSGLT2 in patients with diabetes and established CVD.
**39**	**Pharmacologic treatment**	**Modify**	Reduce the intervals between steps for medication intensification to 2 weeks instead of 1 month.
**40**	**Pharmacologic treatment**	**Modify**	Change the warning "WOMEN of CHILDBEARING AGE" to "WOMEN of CHILDBEARING POTENTIAL"

**41**	**Continuity of care**	**Include**	Recommendation on Home BP measurement for treatment monitoring.

**42**	**Continuity of care**	**Include**	A target time to achieve BP control.

**43**	**Continuity of care**	**Include**	Recommendation of using Telemedicine / mHealth apps to monitor adherence and compliance with recommendations, and to reduce absenteeism.
**44**	**Continuity of care**	**Include**	Recommendation of assessing cognitive function to improve adherence.

**45**	**Continuity of care**	**Include**	Lipid targets in high CVD risk patients.
**46**	**Continuity of care**	**Include**	An advice not to discontinue statin therapy once the control target has been reached.
**47**	**Continuity of care**	**Modify**	Clarify that intensive BP goals only apply to patients <80 years**.**
**48**	**Continuity of care**	**Modify**	BP targets in high CVD risk patients should be BP < 130/80 rather SBP <130.

**49**	**Continuity of care**	**Include**	A contact for patient support groups.
**50**	**Delivery System**	**Include**	Non-physician workers under supervision must follow patients and titrate medication to improve BP control and reduce CVD and mortality.

**51**	**Delivery System**	**Include**	Non-physician workers must perform HTN screening and CVD risk stratification.
**52**	**Delivery System**	**Include**	Non-physician workers must provide counseling on healthy life-style and medication adherence.
**53**	**Vaccines**	**Modify**	Influenza vaccination to all patients with HTN, even those at low and moderate CVD risk.
**54**	**Vaccines**	**Modify**	Indication for pneumococcus vaccination should exclude patients in primary prevention <65 years.
**55**	**Vaccines**	**Modify**	Extend the vaccination to others conditions such as herpes, diphtheria-tetanus or hepatitis**.**

**56**	**System for Monitoring**	**Include**	A message about the importance of registering clinical variables.
**57**	**System for Monitoring**	**Include**	A message about the relevance of having a strategy of preformance evaluation with feedback.

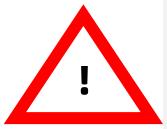

DO NOT FORGET TO FILL THE CONFLICT OF INT
THE SECOND PAGE OF THI

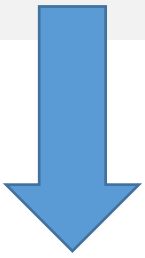

Supporting Evidence	Using the drop-down menu, and regarding the evidence supporting the practice, **determine the appropriateness level for inclusion in the HEARTS Clinical Pathway**. (**1** = lowest confidence; **9** = highest confidence)
CHEP Guidelines Task Force. Hypertension Canada's 2016 Canadian Hypertension Education Program Guidelines for Blood Pressure Measurement, Diagnosis, Assessment of Risk, Prevention, and Treatment of Hypertension. Can J Cardiol. 2016 May;32(5):569-88. doi: 10.1016/j.cjca.2016.02.066.	
2020 WHO technical specifications for automated non-invasive blood pressure measuring devices with cuff. https://www.who.int/publications/i/item/9789240002654	
International Consensus on Standardized Clinic Blood Pressure Measurement - A Call to Action. Am J Med. 2023 May;136(5):438-445.e1. doi: 10.1016/j.amjmed.2022.12.015.	
Blood Pressure Measurement Working Party of the British and Irish Hypertension Society. Measurement of blood pressure in people with atrial fibrillation. J Hum Hypertens. 2019 Nov;33(11):763-765. doi: 10.1038/s41371-019-0261-4. Epub 2019 Oct 2. Erratum in: J Hum Hypertens. 2020 Mar 20	
Lu Y, et a. Simplified blood pressure measurement approaches and implications for hypertension screening: the Atherosclerosis Risk in Communities study. J Hypertens. 2021 Mar 1;39(3):447-452. doi: 10.1097/HJH.0000000000002682.	
Shivashankar R, et al. Validation of a Practical Approach to Blood Pressure Measurement: Secondary Analysis of Data from a Nationally Representative Survey in India. Glob Heart. 2021 Dec 22;16(1):87.	
Brady TM, et al. Effects of Different Rest Period Durations Prior to Blood Pressure Measurement: The Best Rest Trial. Hypertension. 2021 Nov;78(5):1511-1519.	

International Consensus on Standardized Clinic Blood Pressure Measurement - A Call to Action. Am J Med. 2023 May;136(5):438-445.e1. doi: 10.1016/j.amjmed.2022.12.015.	
Schwalm JD, et al. A community-based comprehensive intervention to reduce cardiovascular risk in hypertension (HOPE 4): a cluster-randomised controlled trial. Lancet. 2019 Oct 5;394(10205):1231-1242. doi: 10.1016/S0140-6736(19)31949-X. (2)	
Victor RG, et al. A Cluster-Randomized Trial of Blood-Pressure Reduction in Black Barbershops. N Engl J Med. 2018 Apr 5;378(14):1291-1301. doi: 10.1056/NEJMoa1717250. Epub 2018 Mar 12. PMID: 29527973; PMCID: PMC6018053.	
Guideline for the pharmacological treatment of hypertension in adults. Geneva: World Health Organization; 2021. Licence: CC BY-NC-SA 3.0 IGO.	
Kidney Disease: Improving Global Outcomes (KDIGO) CKD Work Group. KDIGO 2024 Clinical Practice Guideline for the Evaluation and Management of Chronic Kidney Disease. Kidney Int. 2024 Apr;105(4S):S117-S314. doi: 10.1016/j.kint.2023.10.018. PMID: 38490803.	
Garofalo C, et al. Hypertension and prehypertension and prediction of development of decreased estimated GFR in the general population: a meta-analysis of cohort studies.Am J Kidney Dis. 2016; 67:89–97. doi: 10.1053/j.ajkd.2015.08.027	
Writing Group for the CKD Prognosis Consortium. Estimated Glomerular Filtration Rate, Albuminuria, and Adverse Outcomes: An Individual-Participant Data Meta-Analysis. JAMA. 2023 Oct 3;330(13):1266-1277. doi: 10.1001/jama.2023.17002	
Chronic Kidney Disease Prognosis Consortium. Association of estimated glomerular filtration rate and albuminuria with all-cause and cardiovascular mortality in general population cohorts: a collaborative meta-analysis. Lancet. 2010 Jun 12;375(9731):2073–81	
Price CP, et al. Use of protein:creatinine ratio measurements on random urine samples for prediction of significant proteinuria: a systematic review. Clin Chem. 2005 Sep;51(9):1577–86	
Schrader J, et al. Microalbuminuria and tubular proteinuria as risk predictors of cardiovascular morbidity and mortality in essential hypertension: final results of a prospective long-term study (MARPLE Study)*. J Hypertens. 2006 Mar;24(3):541–8	

Karmali KN, et al. Risk scoring for the primary prevention of cardiovascular disease. Cochrane Database Syst Rev. 2017 Mar 14;3(3):CD006887. doi: 10.1002/14651858.CD006887.pub4.	
Kidney Disease: Improving Global Outcomes (KDIGO) CKD Work Group. KDIGO 2024 Clinical Practice Guideline for the Evaluation and Management of Chronic Kidney Disease. Kidney Int. 2024 Apr;105(4S):S117-S314. doi: 10.1016/j.kint.2023.10.018. PMID: 38490803.	
Welton NJ, et al. Screening strategies for atrial fibrillation: a systematic review and cost-effectiveness analysis. Health Technol Assess. 2017 May;21(29):1-236. doi: 10.3310/hta21290.	
European Stroke Organisation (ESO) guideline on screening for subclinical atrial fibrillation after stroke or transient ischaemic attack of undetermined origin. Eur Stroke J. 2022 Sep;7(3):VI. doi: 10.1177/23969873221099478. Epub 2022 Jun 3. Erratum in: Eur Stroke J. 2023 Mar;8(1):413.	
Jin Y, et al. Global burden of atrial fibrillation/flutter due to high systolic blood pressure from 1990 to 2019: estimates from the global burden of disease study 2019. J Clin Hypertens (Greenwich). 2022 Nov;24(11):1461-1472. doi: 10.1111/jch.14584. Epub 2022 Oct 10. PMID: 36210736; PMCID: PMC9659877.	
Verbiest-van Gurp N, et al. Detection of atrial fibrillation in primary care with radial pulse palpation, electronic blood pressure measurement and handheld single-lead electrocardiography: a diagnostic accuracy study. BMJ Open. 2022 Jun 29;12(6):e059172. doi: 10.1136/bmjopen-2021-059172.	
Special Interest Group “Cardiovascular Diseases” of the EuGMS. Atrial fibrillation: a geriatric perspective on the 2020 ESC guidelines. Eur Geriatr Med. 2022 Feb;13(1):5-18. doi: 10.1007/s41999-021-00537-w.	
Kahwati L, et al. Screening for Atrial Fibrillation: An Evidence Review for the U.S. Preventive Services Task Force [Internet]. Rockville (MD): Agency for Healthcare Research and Quality (US); 2022 Jan. Report No.: 21-05277-EF-1.	
Fibrillation Screen, Management, and Guideline-Recommended Therapy in the Rural Primary Care Setting: A Cross-Sectional Study and Cost-Effectiveness Analysis of eHealth Tools to Support All Stages of Screening. J Am Heart Assoc. 2020 Sep 15;9(18):e017080. doi: 10.1161/JAHA.120.017080.	
Screening strategies for atrial fibrillation in the elderly population: a systematic review and network meta-analysis. Clin Res Cardiol. 2023 Jun;112(6):705-715. doi: 10.1007/s00392-022-02117-9	

Moran PS, et al. Systematic screening for the detection of atrial fibrillation. Cochrane Database Syst Rev. 2016 Jun 3;2016(6):CD009586. doi: 10.1002/14651858.CD009586.pub3.	
Taggar JS, et al. Accuracy of methods for detecting an irregular pulse and suspected atrial fibrillation: A systematic review and meta-analysis. Eur J Prev Cardiol. 2016 Aug;23(12):1330-8. doi: 10.1177/2047487315611347.	
Cooke G, et al. Is pulse palpation helpful in detecting atrial fibrillation? A systematic review. J Fam Pract. 2006 Feb;55(2):130-4.	
Zhang W, et al. STEP Study Group. Trial of Intensive Blood-Pressure Control in Older Patients with Hypertension. N Engl J Med. 2021 Sep 30;385(14):1268-1279. doi: 10.1056/NEJMoa2111437.	
Williamson JD, et al. SPRINT Research Group. Intensive vs Standard Blood Pressure Control and Cardiovascular Disease Outcomes in Adults Aged ≥75 Years: A Randomized Clinical Trial. JAMA. 2016 Jun 28;315(24):2673-82. doi: 10.1001/jama.2016.7050.	
Wang Z, et al. The Effect of Frailty on the Efficacy and Safety of Intensive Blood Pressure Control: A Post Hoc Analysis of the SPRINT Trial. Circulation. 2023 Aug 15;148(7):565-574. doi: 10.1161/CIRCULATIONAHA.123.064003.	
Li C, et al. Generalisability and potential deaths averted from intensive blood pressure treatment among the elderly population in the US and China: A nationally representative cross-sectional study. J Glob Health. 2023 Sep 8;13:04100. doi: 10.7189/jogh.13.04100.	
Ho VS, et al. Time to benefit for stroke reduction after blood pressure treatment in older adults: A meta-analysis. J Am Geriatr Soc. 2022 May;70(5):1558-1568. doi: 10.1111/jgs.17684.	
Chapman N, et al. Arterial Hypertension in Women: State of the Art and Knowledge Gaps. Hypertension. 2023 Jun;80(6):1140-1149. doi: 10.1161/HYPERTENSIONAHA.122.20448.	
Hypertension in Pregnancy: Diagnosis, Blood Pressure Goals, and Pharmacotherapy: A Scientific Statement From the American Heart Association. Hypertension. 2022 Feb;79(2):e21-e41. doi: 10.1161/HYP.0000000000000208.	
2023 ESH Guidelines for the management of arterial hypertension The Task Force for the management of arterial hypertension of the European Society of Hypertension: Endorsed by the International Society of Hypertension (ISH) and the European Renal Association (ERA). J Hypertens. 2023 Dec 1;41(12):1874-2071. doi: 10.1097/HJH.0000000000003480. Erratum in: J Hypertens. 2024 Jan 1;42(1):194.	

Yano Y, et al. Association of Blood Pressure Classification in Young Adults Using the 2017 American College of Cardiology/American Heart Association Blood Pressure Guideline With Cardiovascular Events Later in Life. JAMA. 2018 Nov 6;320(17):1774-1782. doi: 10.1001/jama.2018.13551	
Verdecchia P, et al. Prognostic value of a new electrocardiographic method for diagnosis of left ventricular hypertrophy in essential hypertension. J Am Coll Cardiol. 1998 Feb;31(2):383–90.	
Dunn FG, et al. Left ventricular hypertrophy and mortality in hypertension: an analysis of data from the Glasgow Blood Pressure Clinic. J Hypertens. 1990 Aug;8(8):775–82.	
Kim HG, et al. Stress and Heart Rate Variability: A Meta-Analysis and Review of the Literature. Psychiatry Investig. 2018 Mar;15(3):235-245. doi: 10.30773/pi.2017.08.17.	
Li, K.et al. Heart Rate Variability Measurement through a Smart Wearable Device: Another Breakthrough for Personal Health Monitoring? Int. J. Environ. Res. Public Health 2023, 20, 7146. https://doi.org/10.3390/ijerph20247146	
Hillebrand S, et al. Heart rate variability and first cardiovascular event in populations without known cardiovascular disease: meta-analysis and dose-response meta-regression. Europace. 2013 May;15(5):742-9. doi: 10.1093/europace/eus341	
Lin G, et al. Heart rate variability biofeedback decreases blood pressure in prehypertensive subjects by improving autonomic function and baroreflex. J Altern Complement Med. 2012 Feb;18(2):143-52. doi: 10.1089/acm.2010.0607.	
Goldenberg I, et al. Heart Rate Variability for Risk Assessment of Myocardial Ischemia in Patients Without Known Coronary Artery Disease: The HRV-DETECT (Heart Rate Variability for the Detection of Myocardial Ischemia) Study. J Am Heart Assoc. 2019 Dec 17;8(24):e014540. doi: 10.1161/JAHA.119.014540.	
Psychological Health, Well-Being, and the Mind-Heart-Body Connection: A Scientific Statement From the American Heart Association. Circulation. 2021 Mar 9;143(10):e763-e783. doi: 10.1161/CIR.0000000000000947.	

WHO package of essential noncommunicable (PEN) disease interventions for primary health care. https://www.who.int/publications/i/item/9789240009226	
Patel KK, et al. Characteristics and Outcomes of Patients Presenting With Hypertensive Urgency in the Office Setting. JAMA Intern Med. 2016 Jul 1;176(7):981–8.	
Fasce H E, et al. Prevalence of headache, epistaxis, subconjunctival hemorrhages and dizziness in people with normal or high blood pressure. Rev méd Chile. 2002;130(2):160–6.	
2018 ESC/ESH Guidelines for the management of arterial hypertension. Eur Heart J. 2018 Sep 1;39(33):3021–104.	
Bernabe-Ortiz A, et al. Effect of salt substitution on community-wide blood pressure and hypertension incidence. Nat Med. 2020;26(3):374-378. doi:10.1038/s41591-020-0754-2	
Neal B, et al. Effect of Salt Substitution on Cardiovascular Events and Death. N Engl J Med. 2021 Sep 16;385(12):1067-1077. doi: 10.1056/NEJMoa2105675.	
Marklund M, et al. Estimated population wide benefits and risks in China of lowering sodium through potassium enriched salt substitution: modelling study. BMJ. 2020;369:m824. Published 2020 Apr 22. doi:10.1136/bmj.m824;	
Yuan Y, et al. Salt substitution and salt-supply restriction for lowering blood pressure in elderly care facilities: a cluster-randomized trial. Nat Med. 2023 Apr;29(4):973-981. doi: 10.1038/s41591-023-02286-8.	
Zhao X, et al. Using a low-sodium, high-potassium salt substitute to reduce blood pressure among Tibetans with high blood pressure: a patient-blinded randomized controlled trial. PLoS One. 2014;9(10):e110131. Published 2014 Oct 22. doi:10.1371/journal.pone.0110131	

Baffour-Awuah B, et al. Isometric Resistance Training to Manage Hypertension: Systematic Review and Meta-analysis. Curr Hypertens Rep. 2023 Apr;25(4):35-49. doi: 10.1007/s11906-023-01232-w.	
Toward Precision Medicine: Circadian Rhythm of Blood Pressure and Chronotherapy for Hypertension - 2021 NHLBI Workshop Report. Hypertension. 2023 Mar;80(3):503-522. doi: 10.1161/HYPERTENSIONAHA.122.19372.	
Jeffers AM, et al. Association of Cannabis Use With Cardiovascular Outcomes Among US Adults. J Am Heart Assoc. 2024 Mar 5;13(5):e030178. doi: 10.1161/JAHA.123.030178.	
Subramaniam VN, et al. The Cardiovascular Effects of Marijuana: Are the Potential Adverse Effects Worth the High? Mo Med. 2019 Mar-Apr;116(2):146-153.	
Franz CA, Frishman WH. Marijuana Use and Cardiovascular Disease. Cardiol Rev. 2016 Jul-Aug;24(4):158-62. doi: 10.1097/CRD.0000000000000103.	
Skotsimara G, et al. Cardiovascular effects of electronic cigarettes: A systematic review and meta-analysis. Eur J Prev Cardiol. 2019 Jul;26(11):1219-1228. doi: 10.1177/2047487319832975.	
Biswas A, et al. Sedentary Time and Its Association With Risk for Disease Incidence, Mortality, and Hospitalization in Adults. Ann Intern Med. 20 de enero de 2015;162(2):123-32.	
Rezende LFM, et al. All-Cause Mortality Attributable to Sitting Time: Analysis of 54 Countries Worldwide. Am J Prev Med. 1 de agosto de 2016;51(2):253-63.	
Patterson R, et al. Sedentary behaviour and risk of all-cause, cardiovascular and cancer mortality, and incident type 2 diabetes: a systematic review and dose response meta-analysis. Eur J Epidemiol. 1 de septiembre de 2018;33(9):811-29.	
Ekelund U, et al. Dose-response associations between accelerometry measured physical activity and sedentary time and all cause mortality: systematic review and harmonised meta-analysis. BMJ. 21 de agosto de 2019;l4570.	

Phillips EM, et al. The Exercise Prescription: A Tool to Improve Physical Activity. PM&R. 2012;4(11):818-25.	
Arsenijevic J, Groot W. Physical activity on prescription schemes (PARS): do programme characteristics influence effectiveness? Results of a systematic review and meta-analyses. BMJ Open. 1 de febrero de 2017;7(2):e012156.	
Patnode CD, et al. Behavioral Counseling to Promote a Healthful Diet and Physical Activity for Cardiovascular Disease Prevention in Adults Without Known Cardiovascular Disease Risk Factors: Updated Evidence Report and Systematic Review for the US Preventive Services Task Force. JAMA. 2017 Jul 11;318(2):175-193. doi: 10.1001/jama.2017.3303.	
Parati G, et al. Adherence to Single-Pill Versus Free-Equivalent Combination Therapy in Hypertension: A Systematic Review and Meta-Analysis. Hypertension. 2021 Feb;77(2):692-705. doi: 10.1161/HYPERTENSIONAHA.120.15781.	
Mobley CM, et al. Fixed-Dose Combination Medication Use Among US Adults With Hypertension: A Missed Opportunity. J Am Heart Assoc. 2023 Feb 21;12(4):e027486. doi: 10.1161/JAHA.122.027486.	
Derington CG, et al. Antihypertensive Medication Regimens Used by US Adults With Hypertension and the Potential for Fixed-Dose Combination Products: The National Health and Nutrition Examination Surveys 2015 to 2020. J Am Heart Assoc. 2023 Jun 6;12(11):e028573. doi: 10.1161/JAHA.122.028573.	
Rodgers A, et al. Rationale for a New Low-Dose Triple Single Pill Combination for the Treatment of Hypertension. Glob Heart. 2024;19(1):18. Published 2024 Feb 14. doi:10.5334/gh.1283	
Littlejohn TW 3rd, et al. Telmisartan plus amlodipine in patients with moderate or severe hypertension: results from a subgroup analysis of a randomized, placebo-controlled, parallel-group, 4 x 4 factorial study. Postgrad Med. 2009 Mar;121(2):5-14. doi: 10.3810/pgm.2009.03.1972.	

Sung KC, et al. Efficacy and safety of low-dose antihypertensive combination of amlodipine, telmisartan, and chlorthalidone: A randomized, double-blind, parallel, phase II trial. J Clin Hypertens (Greenwich). 2022 Oct;24(10):1298-1309. doi: 10.1111/jch.14570.	
Williams B, et al. British Hypertension Society's PATHWAY Studies Group. Spironolactone versus placebo, bisoprolol, and doxazosin to determine the optimal treatment for drug- resistant hypertension (PATHWAY-2): a randomised, double-blind, crossover trial. Lancet. 2015 Nov 21;386(10008):2059-2068. doi: 10.1016/S0140-6736(15)00257-	
2023 AHA/ACC/ACCP/ASPC/NLA/PCNA Guideline for the Management of Patients With Chronic Coronary Disease: A Report of the American Heart Association/American College of Cardiology Joint Committee on Clinical Practice Guidelines. J Am Coll Cardiol. 2023 Aug 29;82(9):833-955. doi: 10.1016/j.jacc.2023.04.003	
Virani SS, et al. Secondary Prevention for Atherosclerotic Cardiovascular Disease: Comparing Recent US and European Guidelines on Dyslipidemia. Circulation. 2020 Apr 7;141(14):1121-1123. doi: 10.1161/CIRCULATIONAHA.119.044282. Epub 2020 Apr 6. PMID: 32250694.	
Yebyo HG, et al. Comparative effectiveness and safety of statins as a class and of specific statins for primary prevention of cardiovascular disease: A systematic review, meta-analysis, and network meta-analysis of randomized trials with 94,283 participants. Am Heart J. 2019 Apr;210:18-28. doi: 10.1016/j.ahj.2018.12.007.	
Yusuf S, et al. Polypill with or without Aspirin in Persons without Cardiovascular Disease. N Engl J Med. 2021; 384(3): 216-28.	
Roshandel G, et al. Effectiveness of polypill for primary and secondary prevention of cardiovascular diseases (PolyIran): a pragmatic, cluster-randomised trial. Lancet. 2019; 394(10199): 672-683.	
Joseph P, et al. Fixed-dose combination therapies with and without aspirin for primary prevention of cardiovascular disease: an individual participant data meta-analysis. Lancet. 2021; 398(10306): 1133-1146.	

Castellano JM, et al. Polypill Strategy in Secondary Cardiovascular Prevention. N Engl J Med. 2022; 387(11): 967-977.	
Coca A, et al. A practical approach to switch from a multiple pill therapeutic strategy to a polypill-based strategy for cardiovascular prevention in patients with hypertension. J Hypertens 2020; 38; 1890-1898.	
Lopez-Lopez JP, et al. Benefits of the Polypill on Medication Adherence in the Primary and Secondary Prevention of Cardiovascular Disease: A Systematic Review. Vasc Health Risk Manag. 2023; 19: 605-615.	
Agarwal A, et al. Inclusion of Polypills for Prevention of Cardiovascular Disease in the 23rd World Health Organization Model List of Essential Medicines: A Significant Step Towards Reducing Global Cardiovascular Morbidity and Mortality. Global Heart 2024; 19(1).	
Gonzalez-Juanatey JR, et al. The CNIC-Polypill reduces recurrent major cardiovascular events in real-life secondary prevention patients in Spain: The NEPTUNO study. Int J Cardiol. 2022; 361: 116-123.	
Lindson N, et al. Strategies to improve smoking cessation rates in primary care. Cochrane Database Syst Rev. 2021 Sep 6;9(9):CD011556. doi: 10.1002/14651858.CD011556.pub2. PMID: 34693994; PMCID: PMC8543670.	
Rigotti NA, et al. Treatment of Tobacco Smoking: A Review. JAMA. 2022 Feb 8;327(6):566-577. doi: 10.1001/jama.2022.0395.	
Ottawa Model for Smoking Cessation. https://ottawamodel.ottawaheart.ca/sites/ottawamodel.ottawaheart.ca/files/omsc_hmpg/omsc_highlight_document_2016.pdf	
Kidney Disease: Improving Global Outcomes (KDIGO) CKD Work Group. KDIGO 2024 Clinical Practice Guideline for the Evaluation and Management of Chronic Kidney Disease. Kidney Int. 2024 Apr;105(4S):S117-S314. doi:	
The EMPA-KIDNEY Collaborative Group. Empagliflozin in Patients with Chronic Kidney Disease. N Engl J Med. 2023 Jan 12;388(2):117-127. doi: 10.1056/NEJMoa2204233.	

DAPA-CKD Trial Committees and Investigators. Effect of Dapagliflozin on Clinical Outcomes in Patients With Chronic Kidney Disease, With and Without Cardiovascular Disease. Circulation. 2021 Feb 2;143(5):438-448. doi: 10.1161/CIRCULATIONAHA.120.051675.	
Vaduganathan M, et al. SGLT-2 inhibitors in patients with heart failure: a comprehensive meta-analysis of five randomised controlled trials. Lancet. 2022 Sep 3;400(10354):757-767. doi: 10.1016/S0140-6736(22)01429-5	
Palmer SC, et al. Sodium-glucose cotransporter protein-2 (SGLT-2) inhibitors and glucagon-like peptide-1 (GLP-1) receptor agonists for type 2 diabetes: systematic review and network meta-analysis of randomised controlled trials. BMJ. 2021 Jan 13;372:m4573. doi: 10.1136/bmj.m4573. Erratum in: BMJ. 2022 Jan	
McGuire DK, et al. Association of SGLT2 Inhibitors With Cardiovascular and Kidney Outcomes in Patients With Type 2 Diabetes: A Meta-analysis. JAMA Cardiol. 2021 Feb 1;6(2):148-158. doi:	
Lasserson DS, et al. How quickly should we titrate antihypertensive medication? Systematic review modelling blood pressure response from trial data. Heart. 2011 Nov;97(21):1771-5. doi: 10.1136/hrt.2010.221473.	
Canoy D, et al. Blood Pressure Lowering Treatment Trialists' Collaboration; Blood Pressure Lowering Treatment Trialists Collaboration. Antihypertensive drug effects on long-term blood pressure: an individual-level data meta-analysis of randomised clinical trials. Heart. 2022 Jul 27;108(16):1281-1289. doi: 10.1136/heartjnl-2021-320171.	
White BM, et al. Antihypertensive prescribing patterns and hypertension control in females of childbearing age. Am J Health Syst Pharm. 2021 Jul 9;78(14):1317-1322. doi: 10.1093/ajhp/zxab162.	
Wenger NK, et al. Hypertension Across a Woman's Life Cycle. J Am Coll Cardiol. 2018 Apr 24;71(16):1797-1813. doi: 10.1016/j.jacc.2018.02.033.	

Parati G, et al. Home blood pressure monitoring: methodology, clinical relevance and practical application: a 2021 position paper by the Working Group on Blood Pressure Monitoring and Cardiovascular Variability of the European Society of Hypertension. Journal of hypertension. 2021;39:1742-1767	
Acharya S, et al. Self-Measured Blood Pressure-Guided Pharmacotherapy: A Systematic Review and Meta-Analysis of United States-Based Telemedicine Trials. Hypertension. 2024 Mar;81(3):648-657. doi: 10.1161/HYPERTENSIONAHA.123.22109.	
Stergiou GS, et al. 2021 European Society of Hypertension practice guidelines for office and out-of-office blood pressure measurement. Journal of hypertension. 2021;39:1293-1302.	
2017 ACC/AHA/AAPA/ABC/ACPM/AGS/APhA/ASH/ ASPC/ NMA/PCNA Guideline for the Prevention, Detection, Evaluation, and Management of High Blood Pressure in Adults: Executive Summary: A Report of the American College of Cardiology/American Heart Association Task Force on Clinical Practice Guidelines. Hypertension. 2018 Jun;71(6):1269-1324. doi: 10.1161/HYP.0000000000000066. Erratum in: Hypertension. 2018 Jun;71(6):e136-e139. Erratum in: Hypertension. 2018 Sep;72(3):e33	
Parati G, et al. Blood pressure variability: methodological aspects, clinical relevance and practical indications for management - a European Society of Hypertension position paper. Journal of hypertension. 2023; 41(4): 527-544.	
2023 ESH Guidelines for the management of arterial hypertension The Task Force for the management of arterial hypertension of the European Society of Hypertension: Endorsed by the International Society of Hypertension (ISH) and the European Renal Association (ERA). J Hypertens. 2023 Dec 1;41(12):1874-2071. doi: 10.1097/HJH.0000000000003480.	

Status of ambulatory blood pressure monitoring and home blood pressure monitoring for the diagnosis and management of hypertension in the US: an up-to-date review. Hypertens Res. 2023 Mar;46(3):620-629. doi: 10.1038/s41440-022-01137-2	
HCPIA Investigators. Effect of a Community Health Worker-Led Multicomponent Intervention on Blood Pressure Control in Low-Income Patients in Argentina: A Randomized Clinical Trial. JAMA. 2017 Sep 19;318(11):1016-1025. doi: 10.1001/jama.2017.11358.	
Campbell NRC, et al. 2021 World Health Organization guideline on pharmacological treatment of hypertension: Policy implications for the region of the Americas. Lancet Reg Health Am. 2022;9:100219.	
American Heart Association and American Medical Association. Implementation Strategies to Improve Blood Pressure Control in the United States: A Scientific Statement From the American Heart Association and American Medical Association. Hypertension. 2023 Oct;80(10):e143-e157. doi: 10.1161/HYP.0000000000000232.	
Beratarrechea A, et al. Using mH ealth Tools to Improve Access and Coverage of People With Public Health Insurance and High Cardiovascular Disease Risk in Argentina: A Pragmatic Cluster Randomized Trial. J Am Heart Assoc. 2019 Apr 16;8(8):e011799. doi: 10.1161/JAHA.118.011799.	
Boima V, et al. Effectiveness of digital health interventions on blood pressure control, lifestyle behaviours and adherence to medication in patients with hypertension in low-income and middle-income countries: a systematic review and meta-analysis of randomised controlled trials. EClinicalMedicine. 2024 Feb 1;69:102432. doi: 10.1016/j.eclinm.2024.102432.	
DigiCare4You Consortium. Effectiveness, reach, uptake, and feasibility of digital health interventions for adults with hypertension: a systematic review and meta-analysis of randomised controlled trials. Lancet Digit Health. 2023 Mar;5(3):e144-e159. doi: 10.1016/S2589-7500(23)00002-X.	

Kassavou A, et al. The Association Between Smartphone App-Based Self-monitoring of Hypertension-Related Behaviors and Reductions in High Blood Pressure: Systematic Review and Meta-analysis. JMIR Mhealth Uhealth. 2022 Jul 12;10(7):e34767. doi: 10.2196/34767	
Xu H, Long H. The Effect of Smartphone App-Based Interventions for Patients With Hypertension: Systematic Review and Meta-Analysis. JMIR Mhealth Uhealth. 2020 Oct 19;8(10):e21759. doi:	
Kario K, et al. Efficacy of a digital therapeutics system in the management of essential hypertension: the HERB-DH1 pivotal trial. Eur Heart J. 2021 Oct 21;42(40):4111-4122	
McManus RJ, et al. Home and Online Management and Evaluation of Blood Pressure (HOME BP) using a digital intervention in poorly controlled hypertension: randomised controlled trial. BMJ 2021; 372:m4858.	
Omboni S, et al. Evidence and Recommendations on the Use of Telemedicine for the Management of Arterial Hypertension: An International Expert Position Paper. Hypertension. 2020 Nov;76(5):1368-1383. doi: 10.1161/HYPERTENSIONAHA.120.15873.	
Khan NA, et al. Virtual management of hypertension: lessons from the COVID-19 pandemic-International Society of Hypertension position paper endorsed by the World Hypertension League and European Society of Hypertension. J Hypertens. 2022 Aug 1;40(8):1435-1448. doi: 10.1097/HJH.0000000000003205.	
Livingston G, et al. Dementia prevention, intervention, and care: 2020 report of the Lancet Commission. Lancet. 2020 Aug 8;396(10248):413-446. doi: 10.1016/S0140-6736(20)30367-6. Erratum in: Lancet. 2023 Sep 30;402(10408):1132.	

ESC Scientific Document Group. 2016 European Guidelines on cardiovascular disease prevention in clinical practice: The Sixth Joint Task Force of the European Society of Cardiology and Other Societies on Cardiovascular Disease Prevention in Clinical Practice (constituted by representatives of 10 societies and by invited experts)Developed with the special contribution of the European Association for Cardiovascular Prevention & Rehabilitation (EACPR). Eur Heart J. 2016 Aug 1;37(29):2315-2381. doi: 10.1093/eurheartj/ehw106.	
Shah NP, et al. Bending the Cardiovascular Event Curve by Evaluating the Potential Impact of Achieving Low-Density Lipoprotein Cholesterol Goal Across a Large Health System Among Secondary Prevention Patients. Am J Cardiol. 2023 Jan 1;186:91-99. doi: 10.1016/j.amjcard.2022.10.033.	
AHA/ACC/AACVPR/AAPA/ABC/ACPM/ADA/AGS/APhA/ASPC/NLA/PCNA Guideline on the Management of Blood Cholesterol: Executive Summary: A Report of the American College of Cardiology/American Heart Association Task Force on Clinical Practice Guidelines. J Am Coll Cardiol. 2019 Jun 25;73(24):3168-3209. doi: 10.1016/j.jacc.2018.11.002. Erratum in: J Am Coll Cardiol. 2019 Jun 25;73(24):3234-3237.	
HYVET Study Group. Treatment of hypertension in patients 80 years of age or older. N Engl J Med. 2008 May 1;358(18):1887-98. doi: 10.1056/NEJMoa0801369.	
Pajewski NM, et al. Intensive vs Standard Blood Pressure Control in Adults 80 Years or Older: A Secondary Analysis of the Systolic Blood Pressure Intervention Trial. J Am Geriatr Soc. 2020 Mar;68(3):496-504. doi: 10.1111/jgs.16272.	
Not provided.	

Sanya RE, et al. Effectiveness of self-financing patient-led support groups in the management of hypertension and diabetes in low- and middle-income countries: Systematic review. Trop Med Int Health. 2023 Feb;28(2):80-89. doi: 10.1111/tmi.13842.	
Sherifali D, et al. Peer Support for Type 2 Diabetes Management in Low- and Middle-Income Countries (LMICs): A Scoping Review. Glob Heart. 2024 Feb 20;19(1):20. doi: 10.5334/gh.1299.	
Otieno P, et al. Effect of Patient Support Groups for Hypertension on Blood Pressure among Patients with and Without Multimorbidity: Findings from a Cohort Study of Patients on a Home-Based Self-Management Program in Kenya. Glob Heart. 2023 Jun 9;18(1):28. doi: 10.5334/gh.1208.	
Garrison TA, et al. Effect of Occupational Therapy in Promoting Medication Adherence in Primary Care: A Randomized Controlled Trial. Am J Occup Ther. 2023 May 1;77(3):7703205040. doi: 10.5014/ajot.2023.050109.	
CRHCP Study Group. Effectiveness of a non-physician community health-care provider-led intensive blood pressure intervention versus usual care on cardiovascular disease (CRHCP): an open-label, blinded-endpoint, cluster-randomised trial. Lancet. 2023 Mar 18;401(10380):928-938. doi: 10.1016/S0140-6736(22)02603-4.	
Campbell NRC, et al. 2021 World Health Organization guideline on pharmacological treatment of hypertension: Policy implications for the region of the Americas. Lancet Reg Health Am. 2022 May;9:None. doi: 10.1016/j.lana.2022.100219.	
CRHCP Study Group. Effectiveness of a non-physician community health-care provider-led intensive blood pressure intervention versus usual care on cardiovascular disease (CRHCP): an open-label, blinded-endpoint, cluster-randomised trial. Lancet. 2023 Mar 18;401(10380):928-938. doi: 10.1016/S0140-6736(22)02603-4.	

HCPIA Investigators. Effect of a Community Health Worker-Led Multicomponent Intervention on Blood Pressure Control in Low-Income Patients in Argentina: A Randomized Clinical Trial. JAMA. 2017 Sep 19;318(11):1016-1025. doi: 10.1001/jama.2017.11358.	
Beratarrechea A, et al. Using mH ealth Tools to Improve Access and Coverage of People With Public Health Insurance and High Cardiovascular Disease Risk in Argentina: A Pragmatic Cluster Randomized Trial. J Am Heart Assoc. 2019 Apr 16;8(8):e011799. doi: 10.1161/JAHA.118.011799.	
Anand TN, et al. Task sharing with non-physician health-care workers for management of blood pressure in low-income and middle-income countries: a systematic review and meta-analysis. Lancet Glob Health. 2019 Jun;7(6):e761-e771. doi: 10.1016/S2214-109X(19)30077-4. Erratum in: Lancet Glob Health. 2019 Nov;7(11):e1499.	
Maria JL, et al. Task-sharing interventions for improving control of diabetes in low-income and middle-income countries: a systematic review and meta-analysis. Lancet Glob Health. 2021 Feb;9(2):e170-e180. doi: 10.1016/S2214-109X(20)30449-6.	
CDC. (2023, November 16). Adult immunization schedule - healthcare providers| CDC. Centers for Disease Control and Prevention. https://www.cdc.gov/vaccines/schedules/hcp/imz/adult-index.html.	
Kobayashi M, et al. Pneumococcal Vaccine for Adults Aged ≥19 Years: Recommendations of the Advisory Committee on Immunization Practices, United States, 2023. MMWR Recomm Rep. 2023 Sep 8;72(3):1-39. doi: 10.15585/mmwr.rr7203a1.	
CDC. (2023, November 16). Adult immunization schedule - healthcare providers| CDC. Centers for Disease Control and Prevention. https://www.cdc.gov/vaccines/schedules/hcp/imz/adult-index.html.	
Prado P, et al. Monitoring and evaluation platform for HEARTS in the Americas: improving population-based hypertension control programs in primary health care. Rev Panam Salud Publica. 2022 Sep 16;46:e161. doi: 10.26633/RPSP.2022.161.	

Behling EM, et al. Improvement in Hypertension Control Among Adults Seen in Federally Qualified Health Center Clinics in the Stroke Belt: Implementing a Program with a Dashboard and Process Metrics. Health Equity. 2023 Feb 8;7(1):89-99. doi: 10.1089/heq.2022.0109.	
Sim JJ, et al. Systemic implementation strategies to improve hypertension: the Kaiser Permanente Southern California experience. Can J Cardiol. 2014 May;30(5):544-52. doi: 10.1016/j.cjca.2014.01.003.	
Ordunez P, et al. HEARTS in the Americas: Targeting Health System Change to Improve Population Hypertension Control. Curr Hypertens Rep. 2024 Apr;26(4):141-156. doi: 10.1007/s11906-023-01286-w.	
Brettler JW, et al. Drivers and scorecards to improve hypertension control in primary care practice: Recommendations from the HEARTS in the Americas Innovation Group. Lancet Reg Health Am. 2022 May;9:None. doi: 10.1016/j.lana.2022.100223.	

EREST FORM INCLUDED IN S FILE

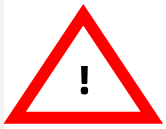

Additional Comments

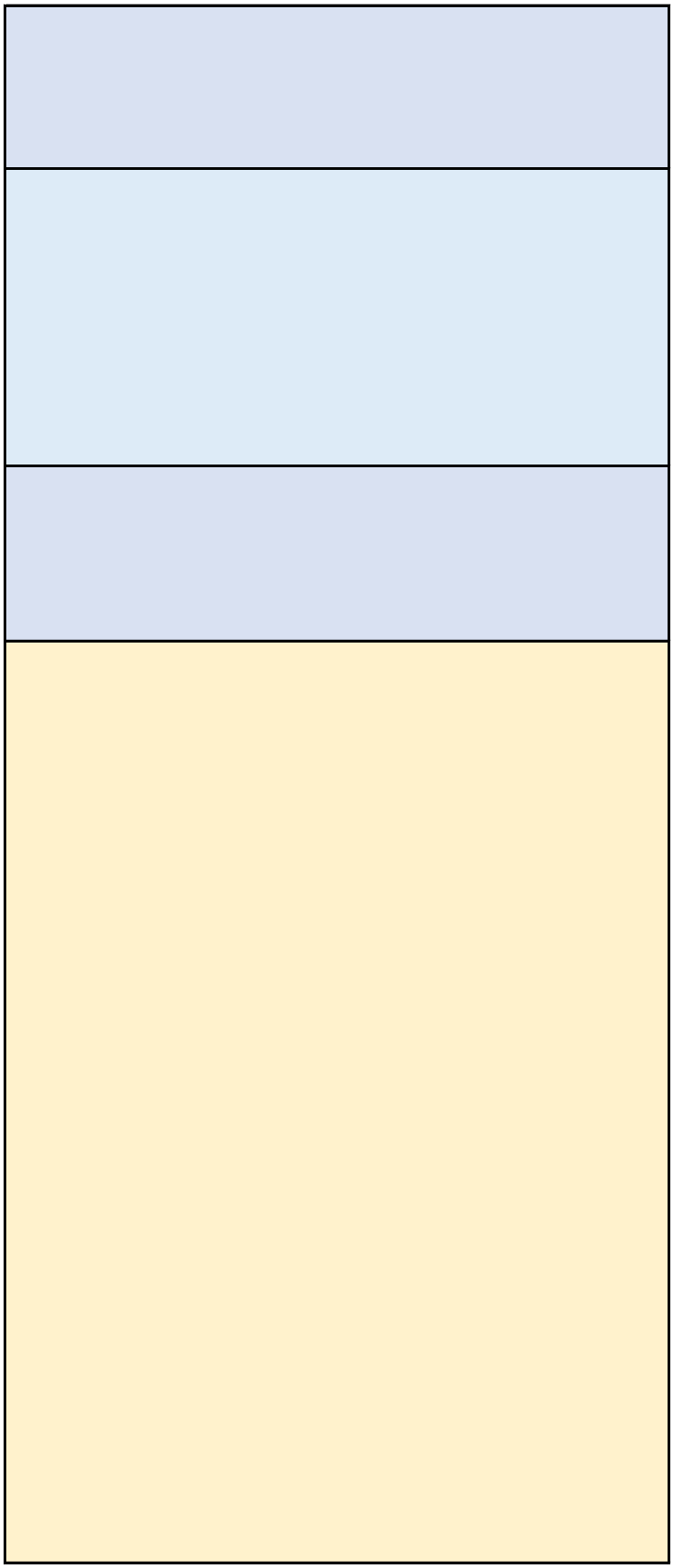

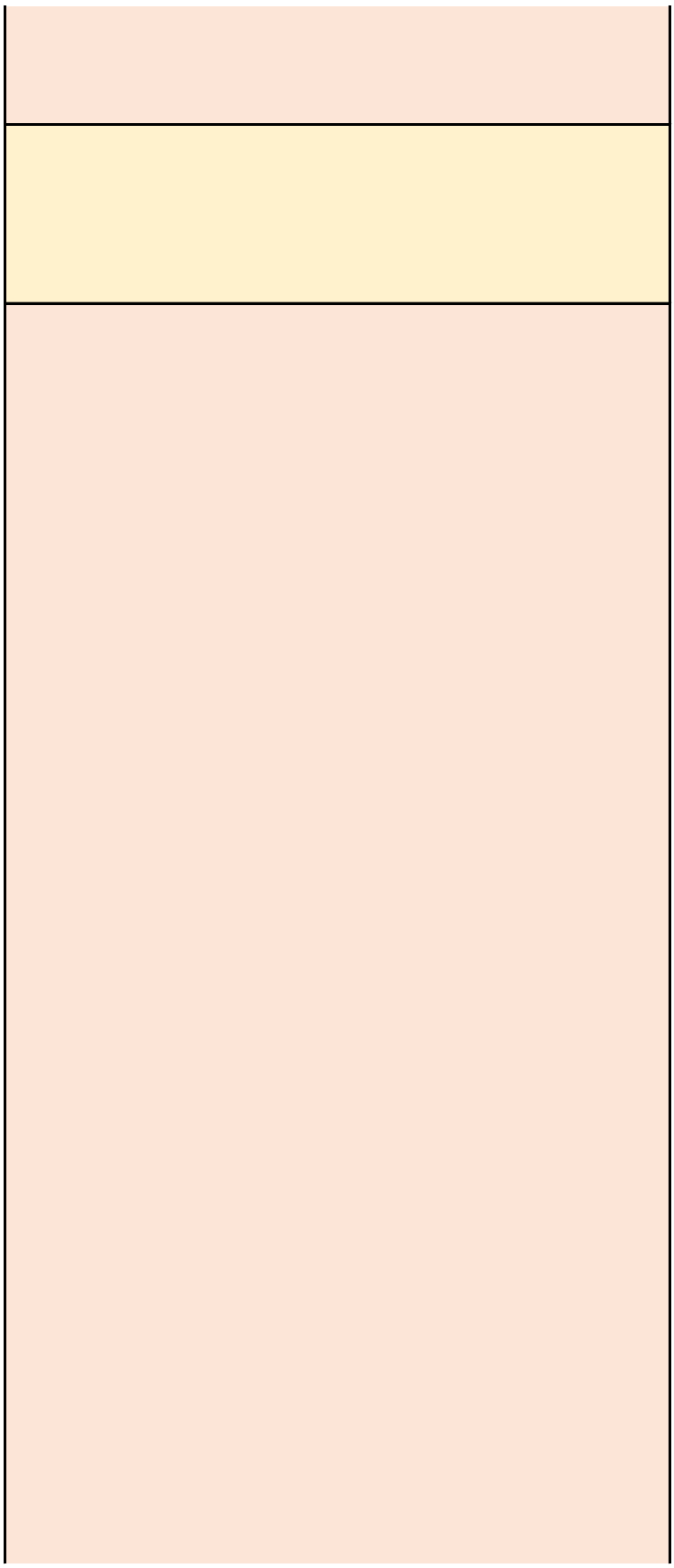

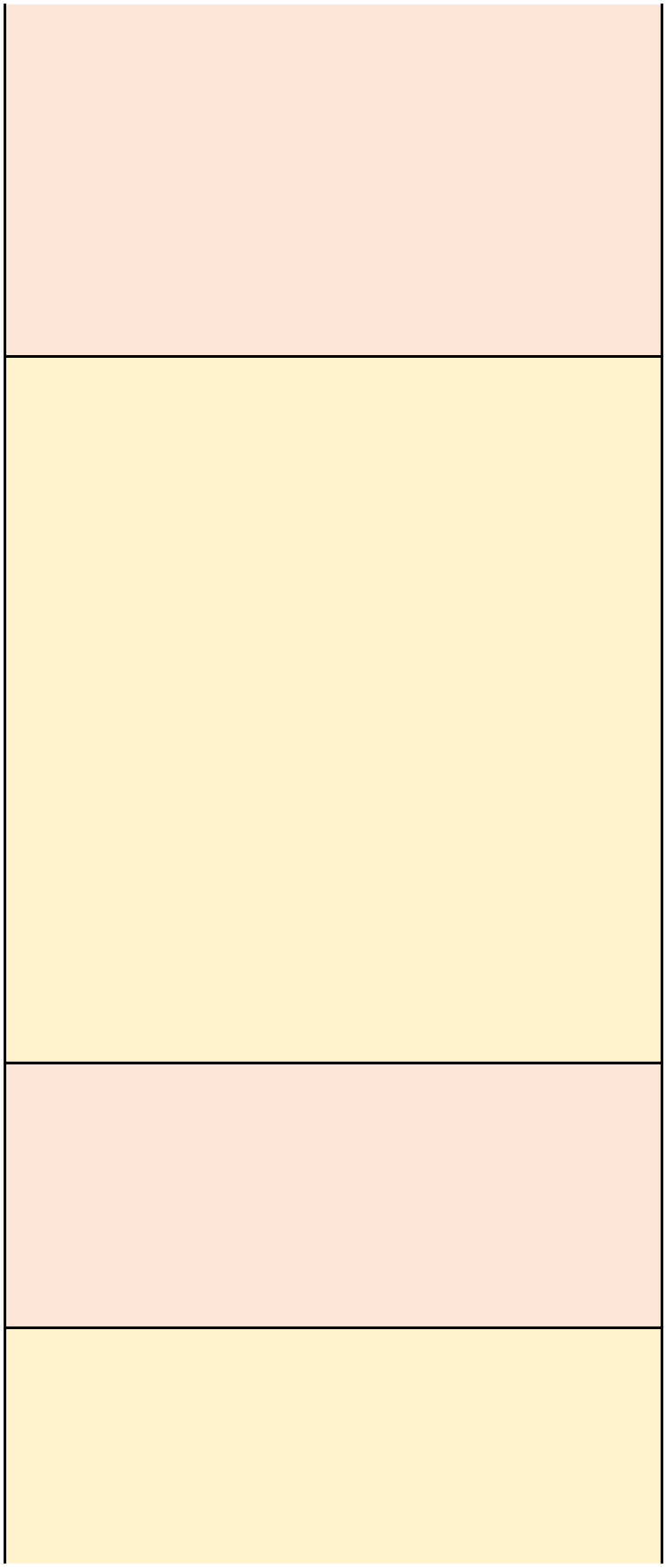

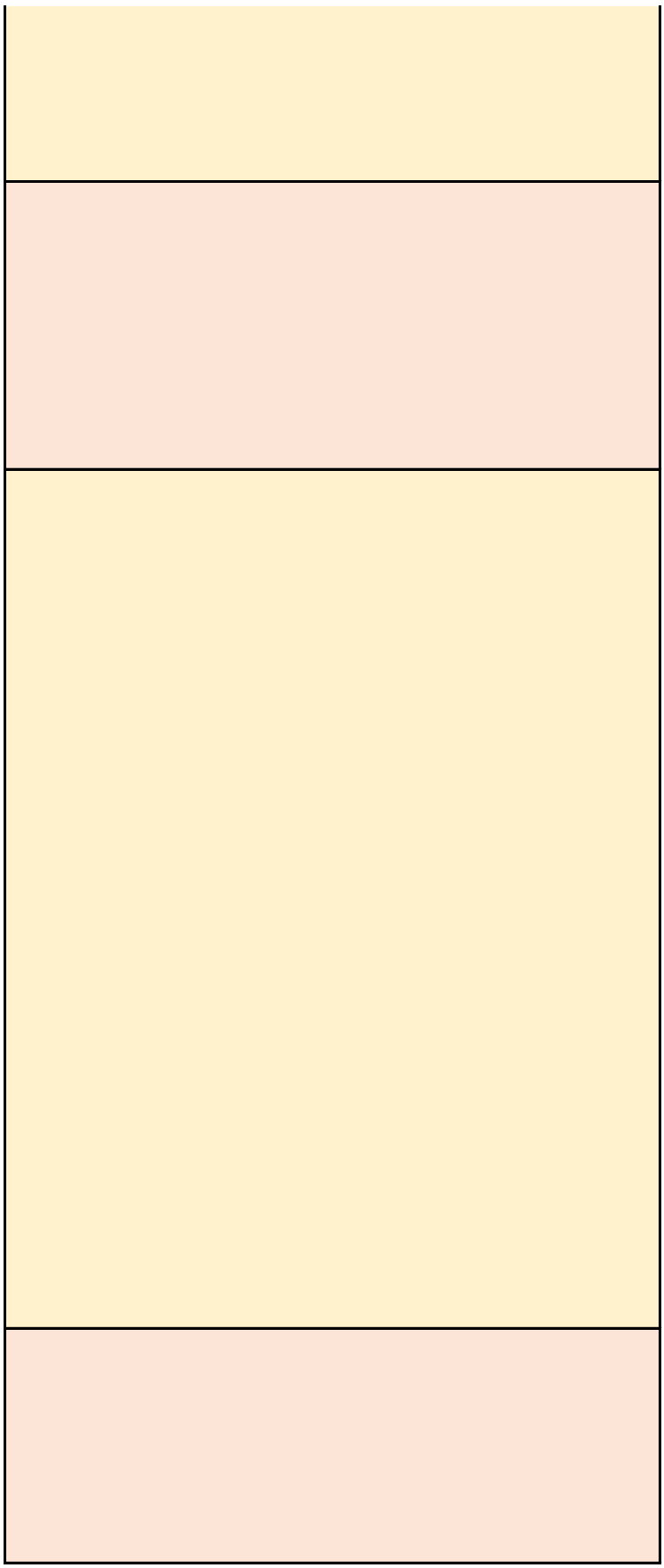

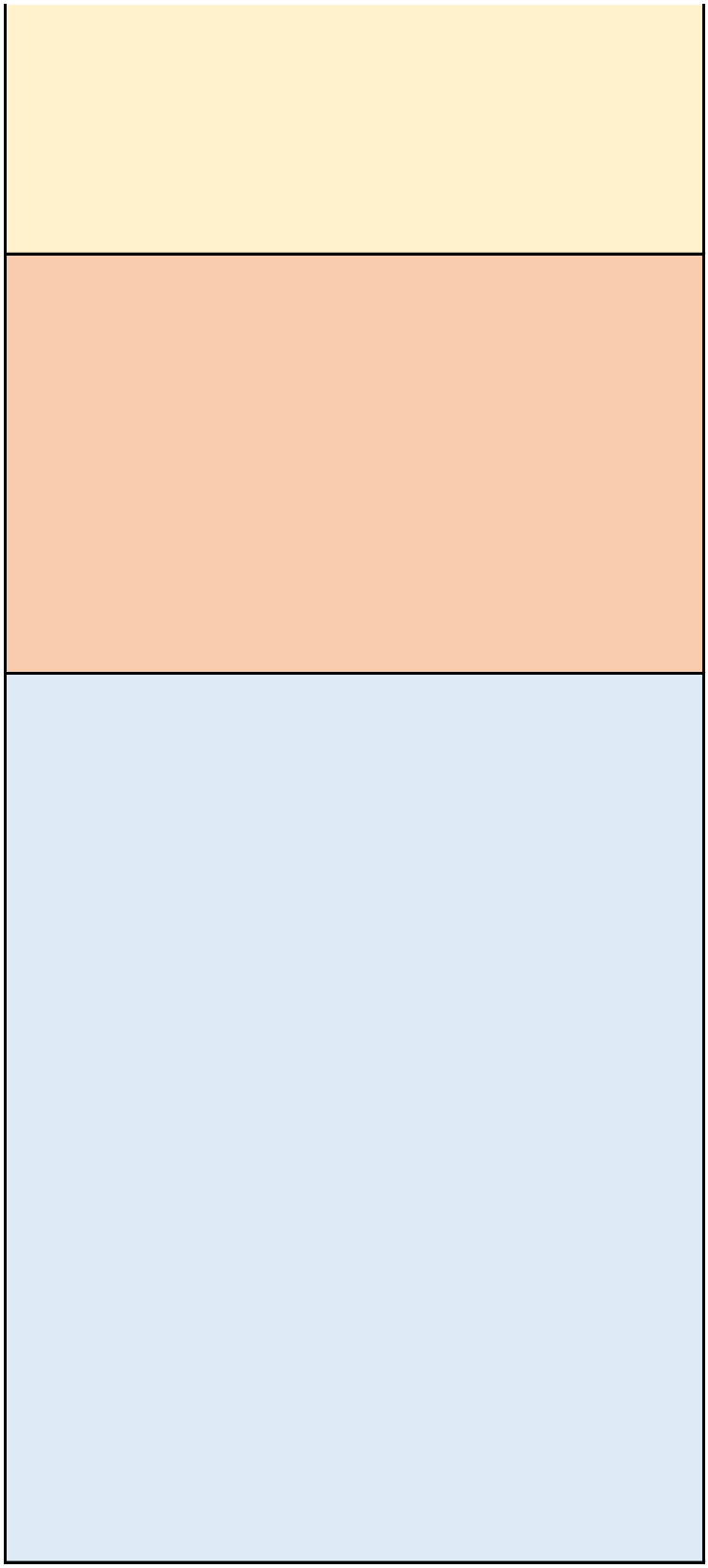

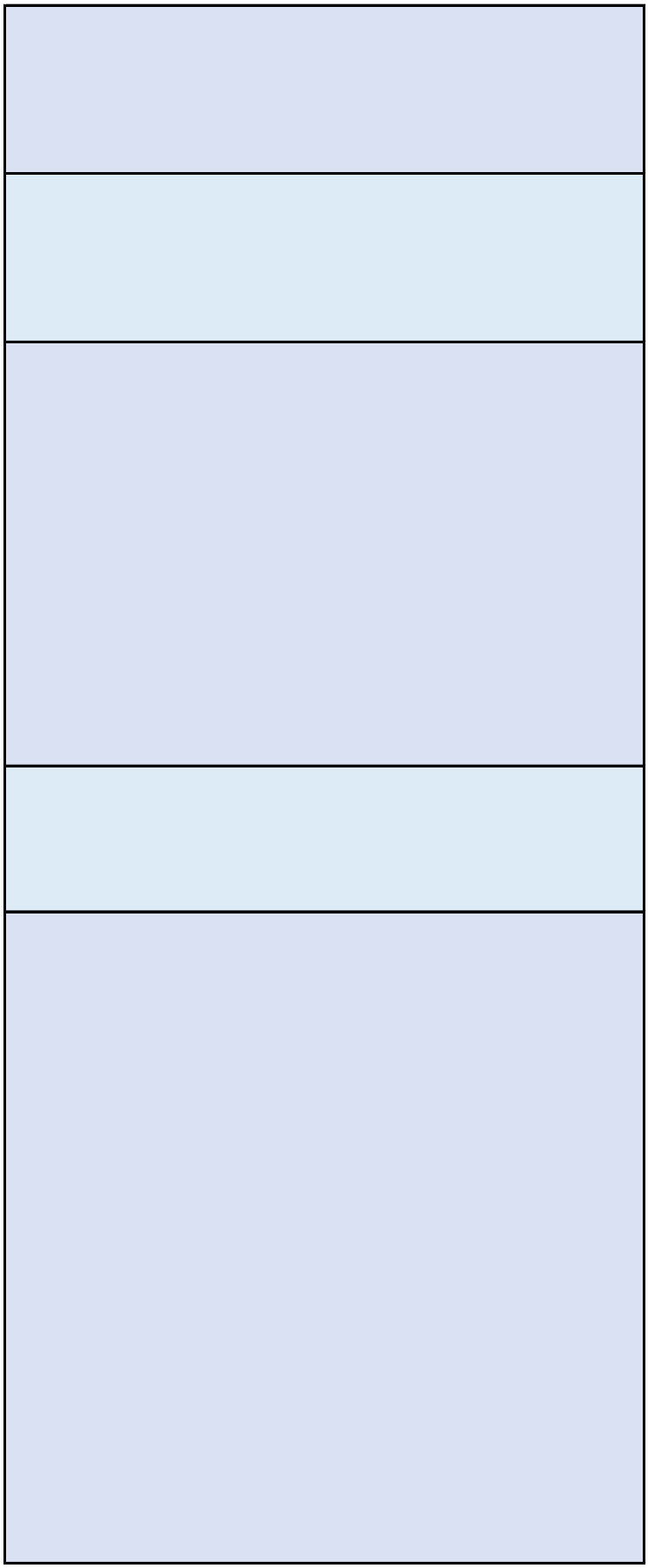

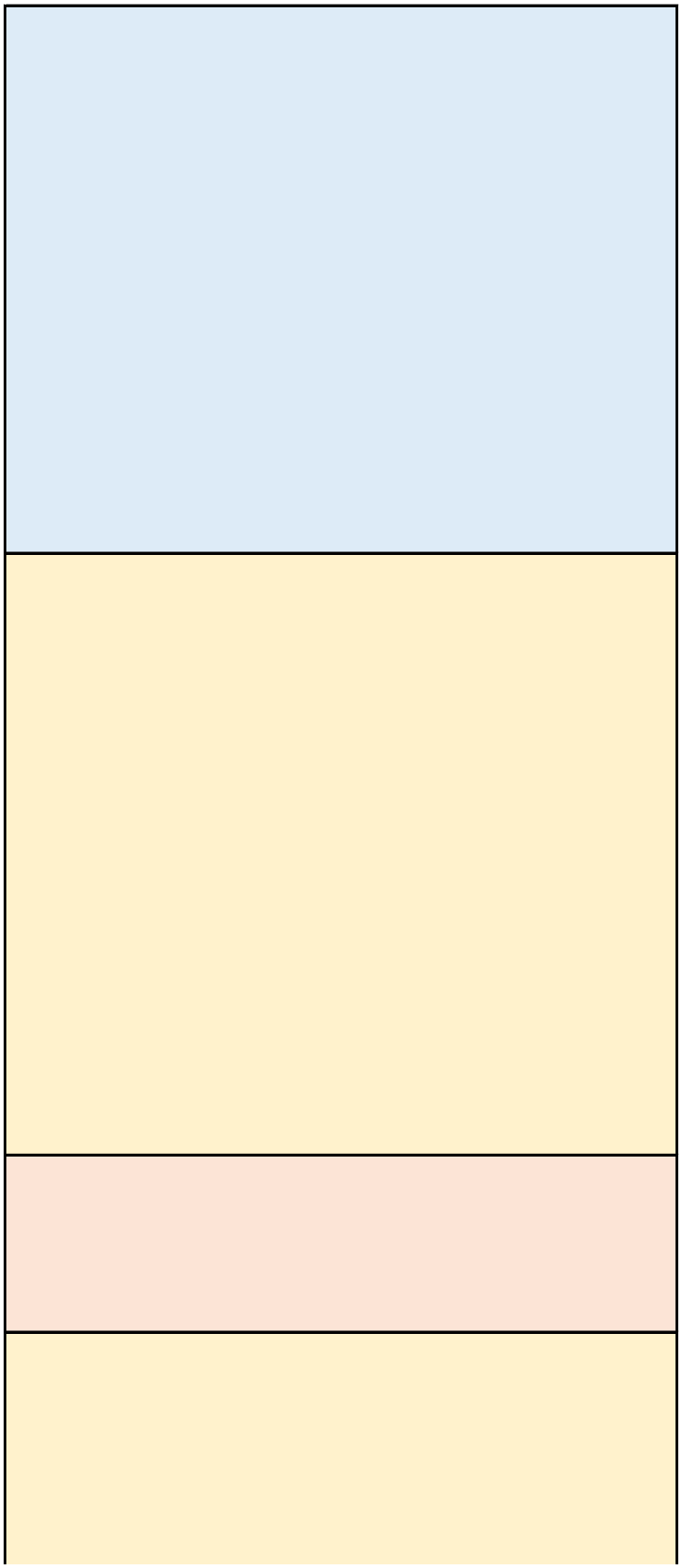

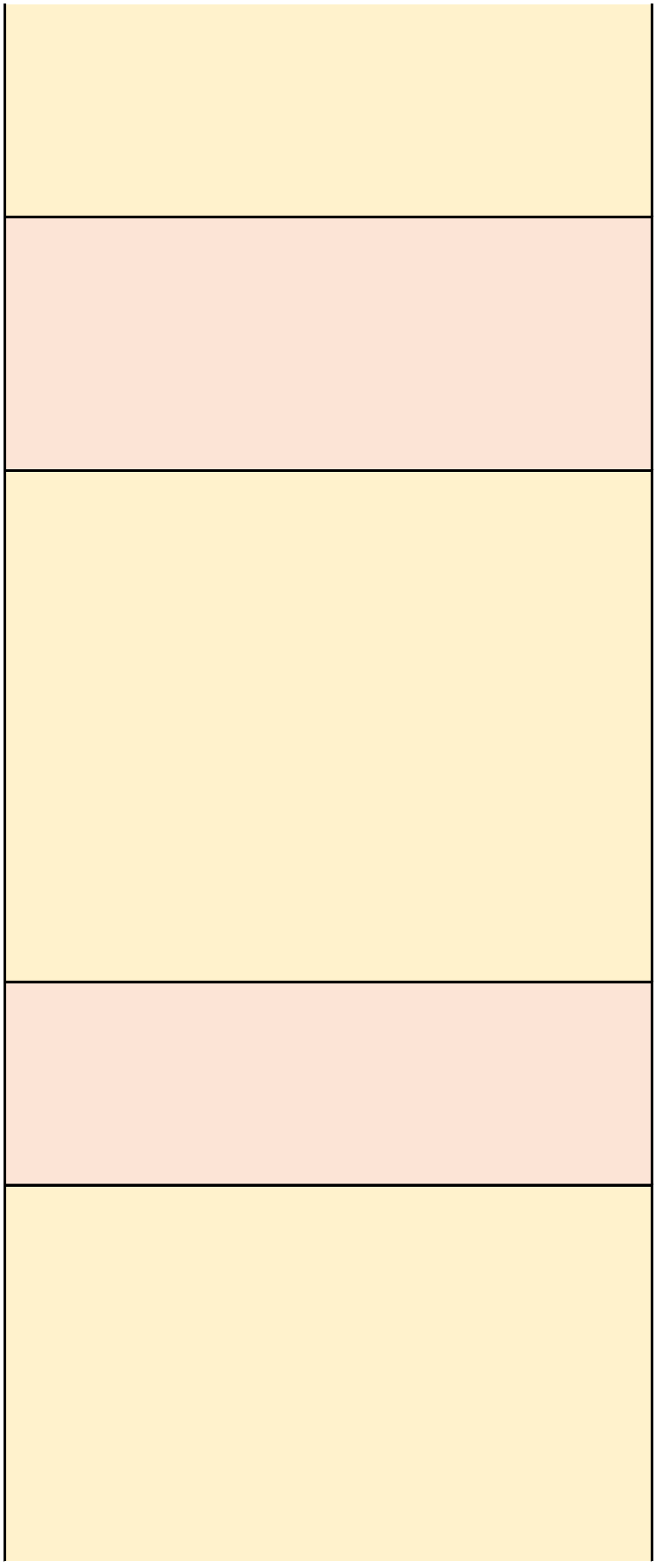

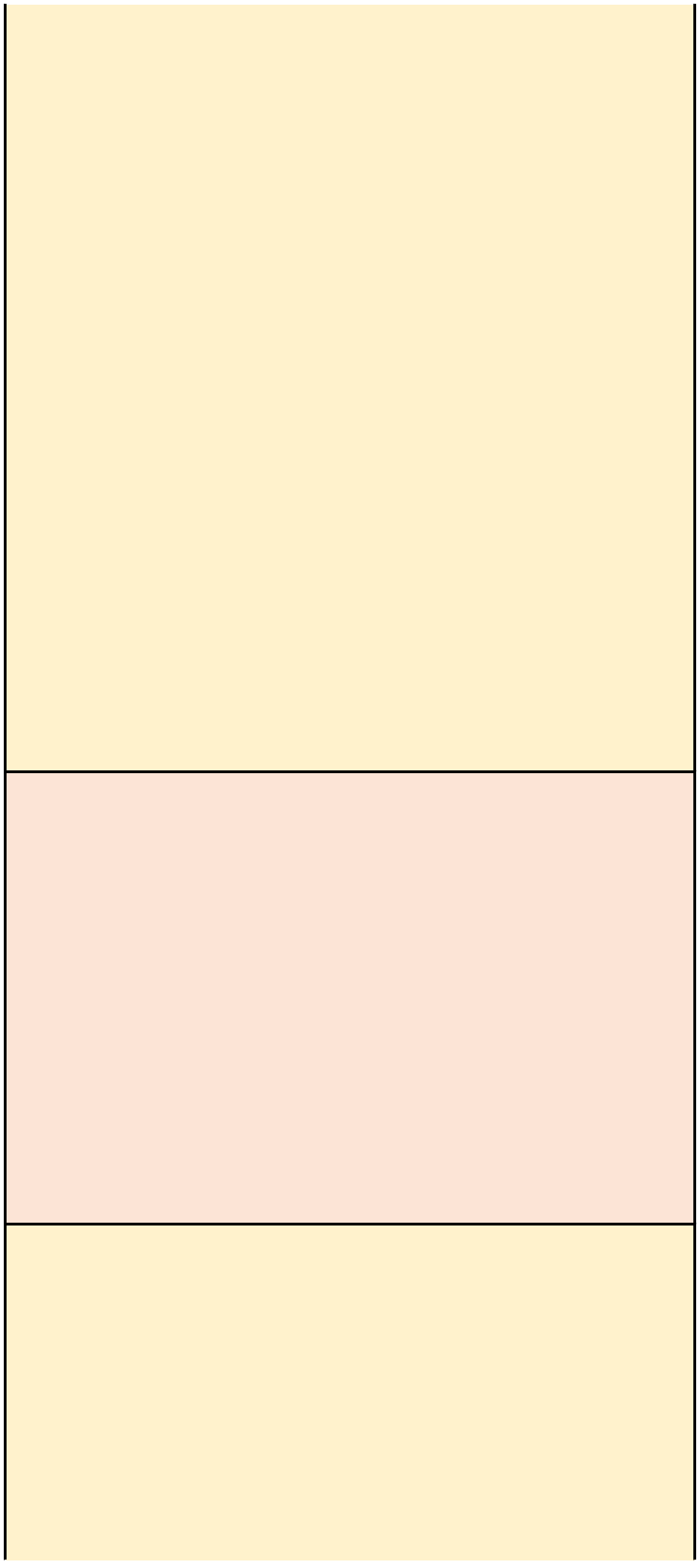

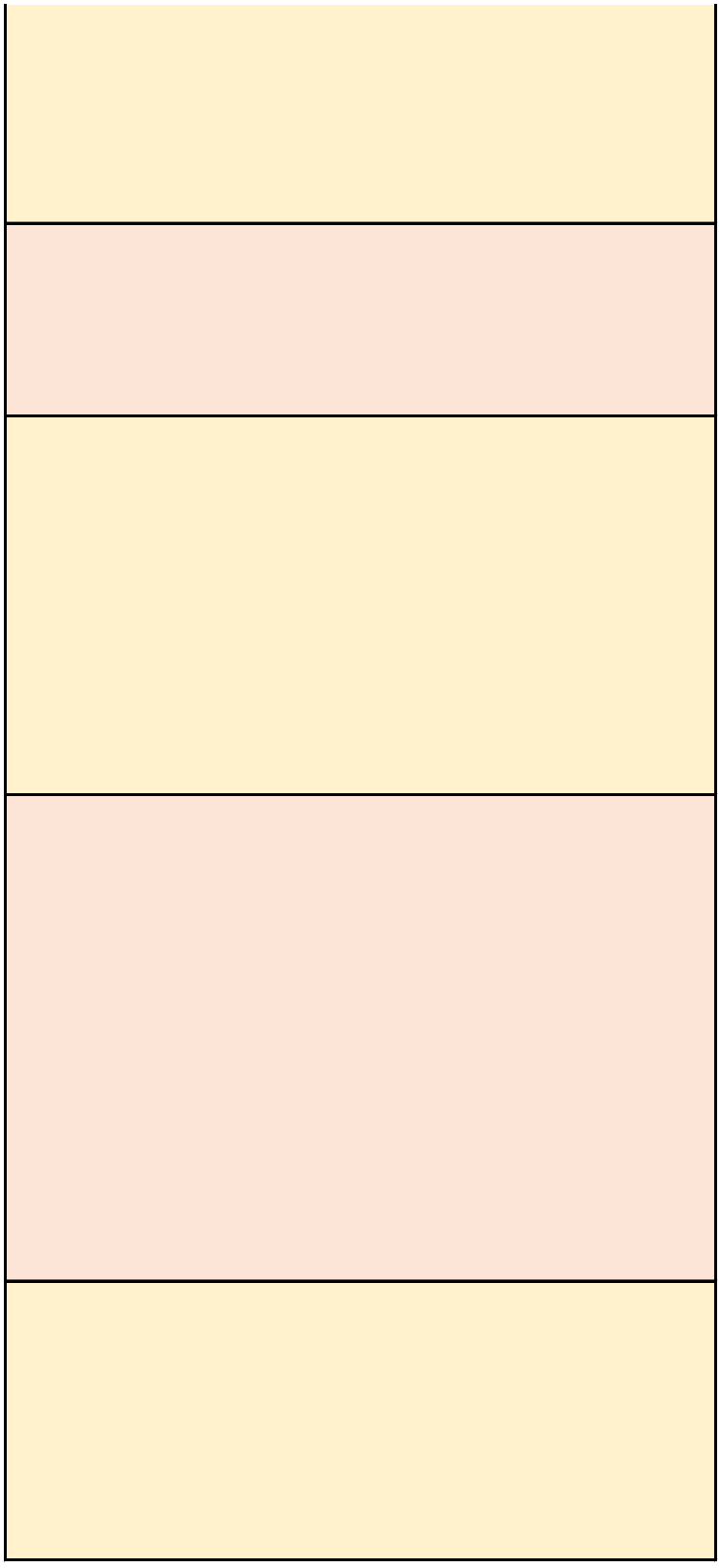

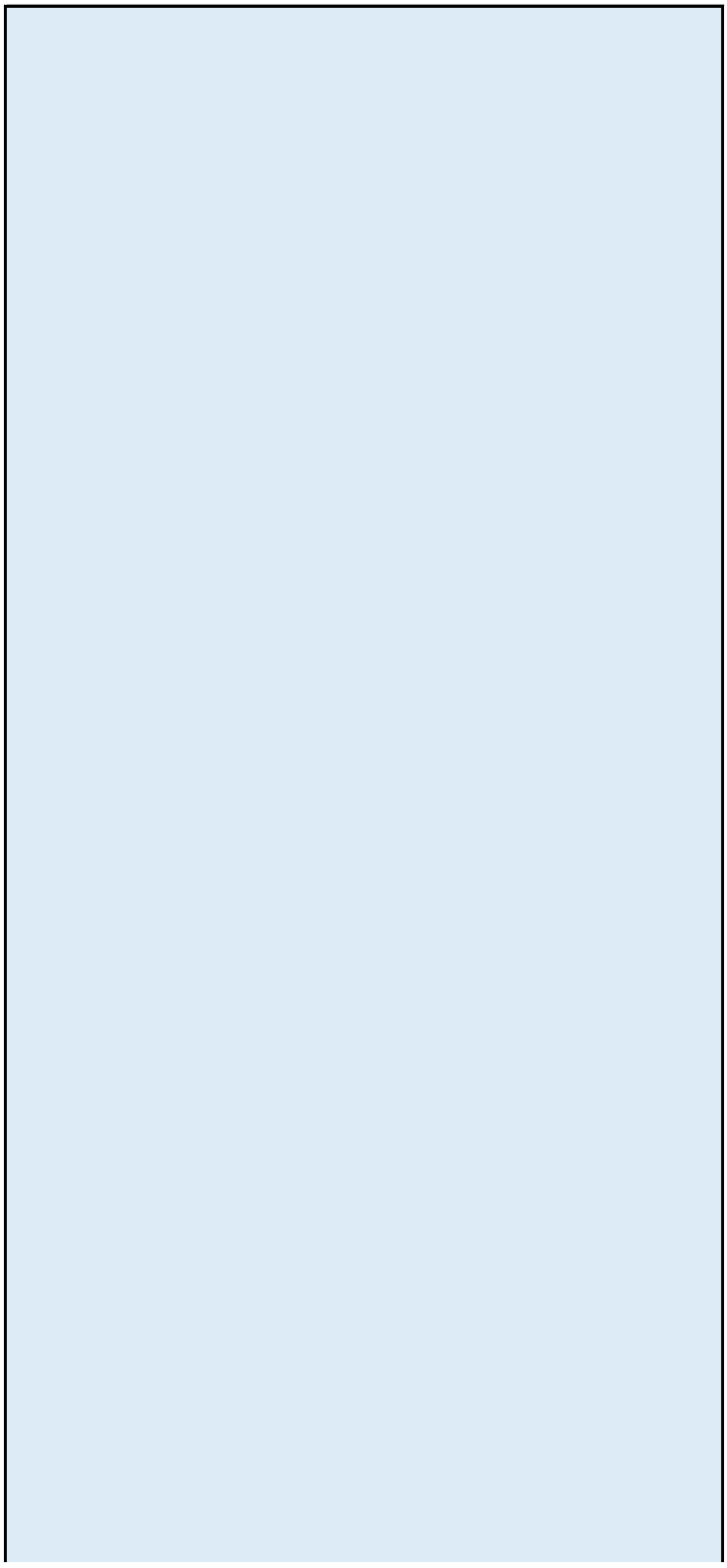

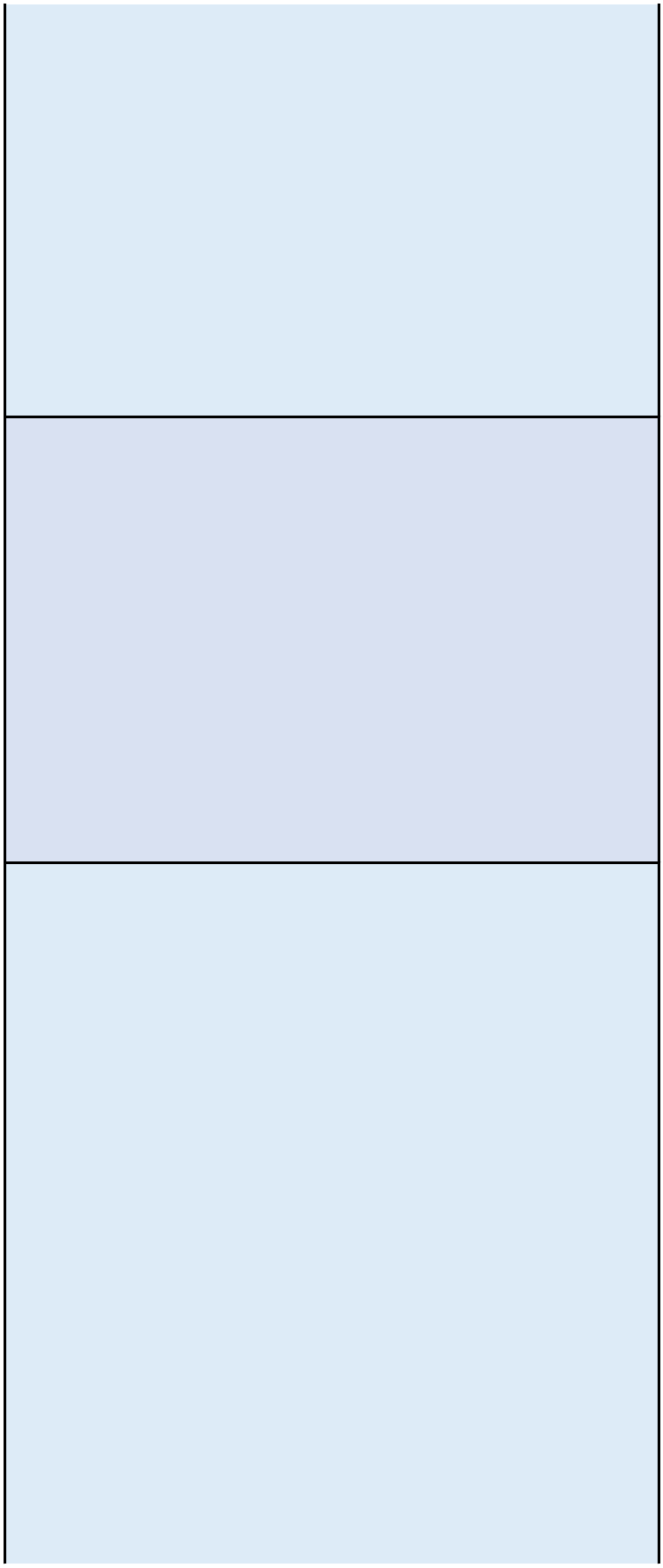

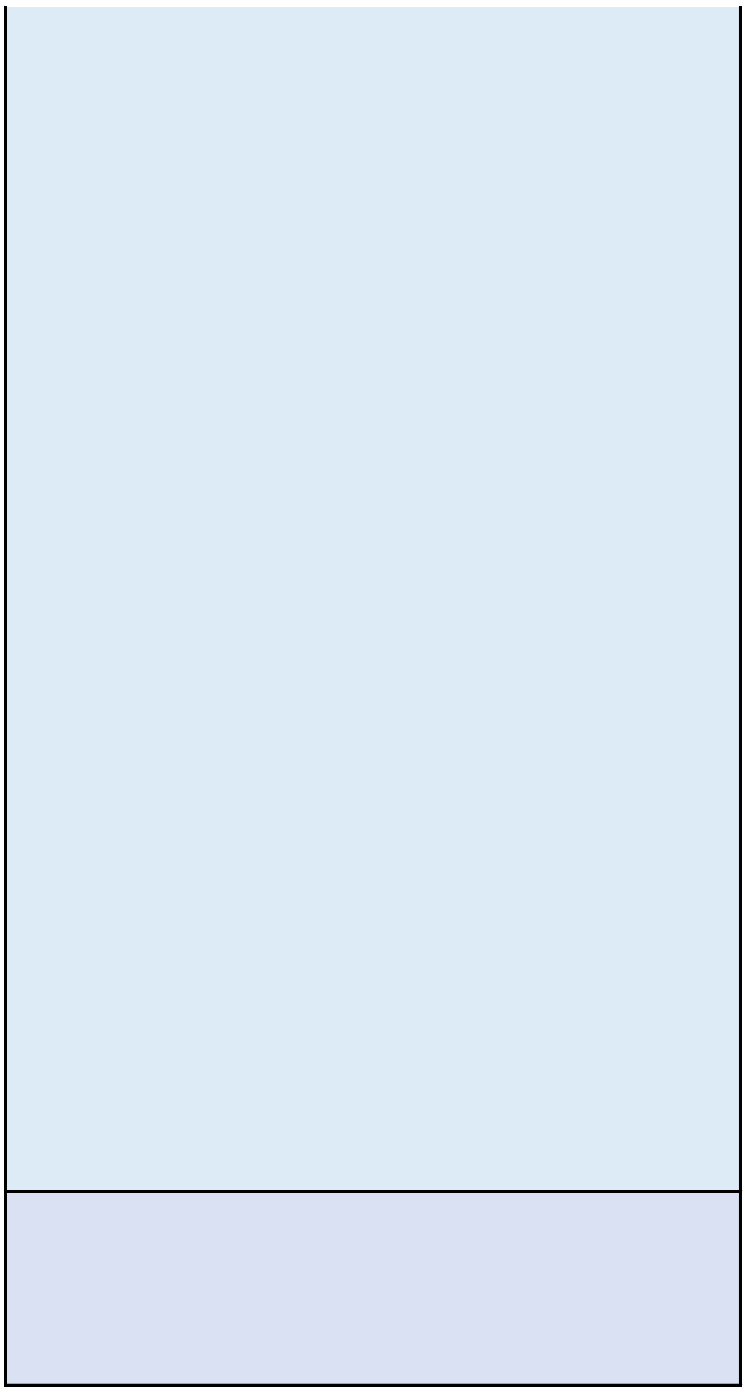

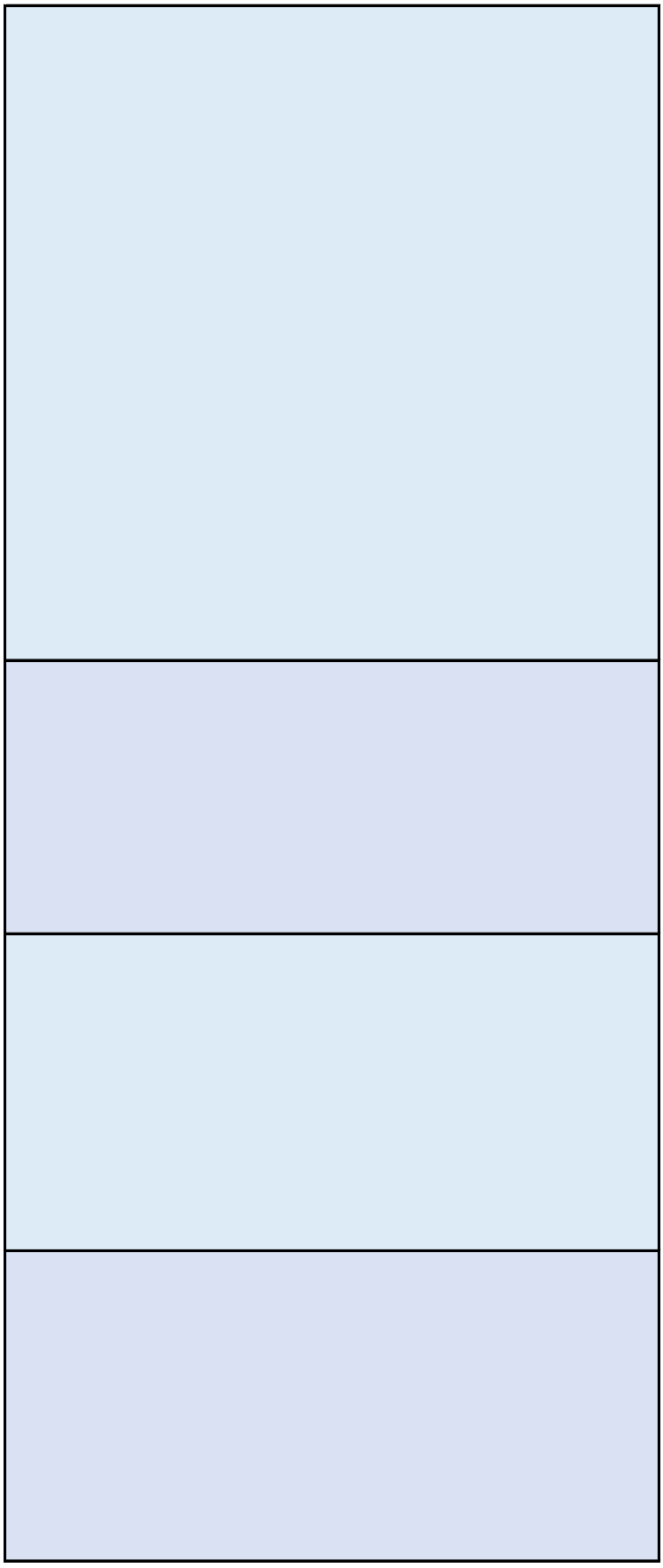

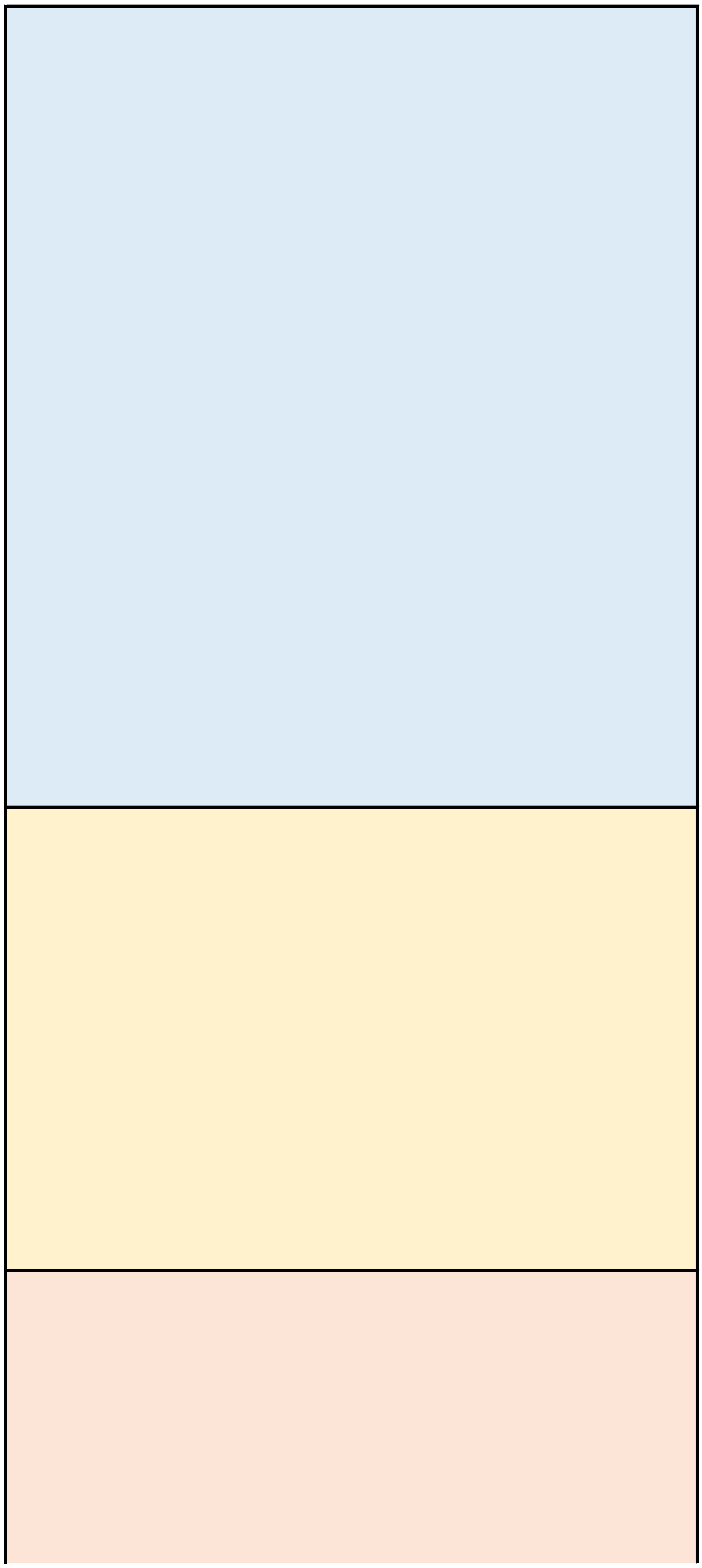

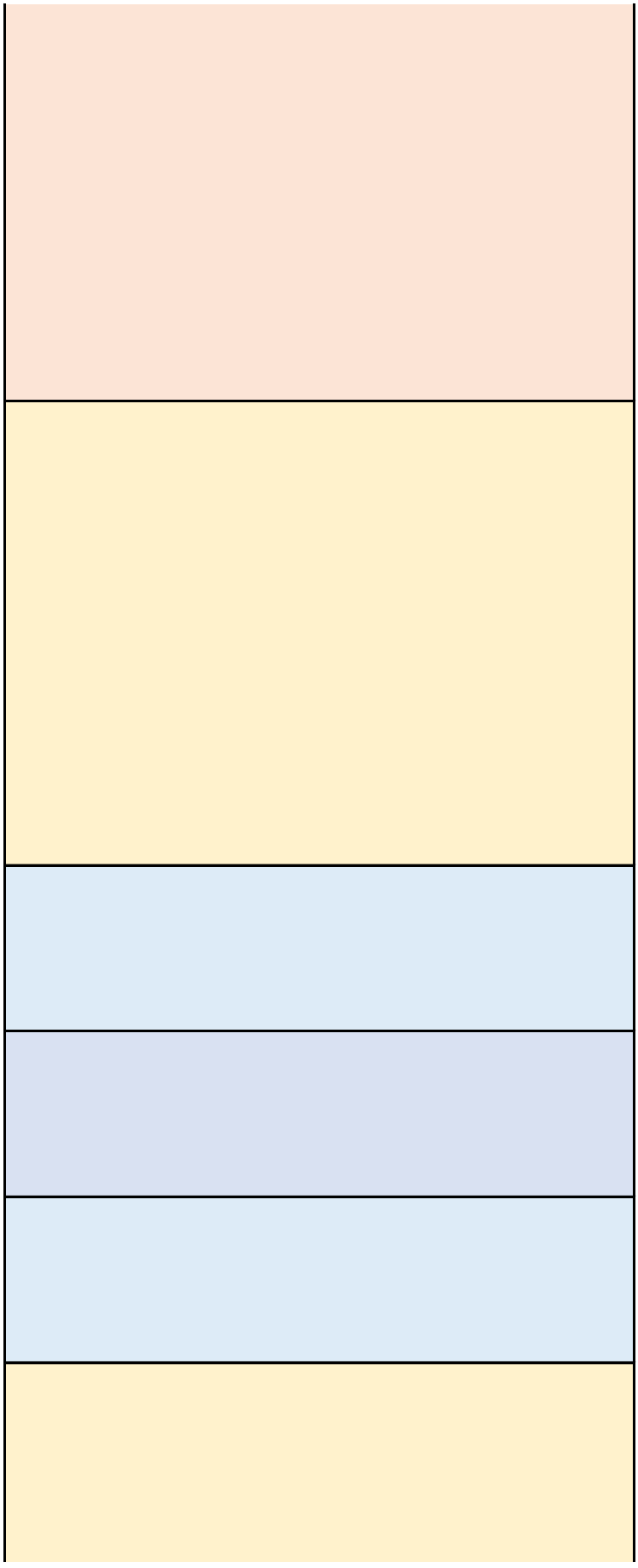

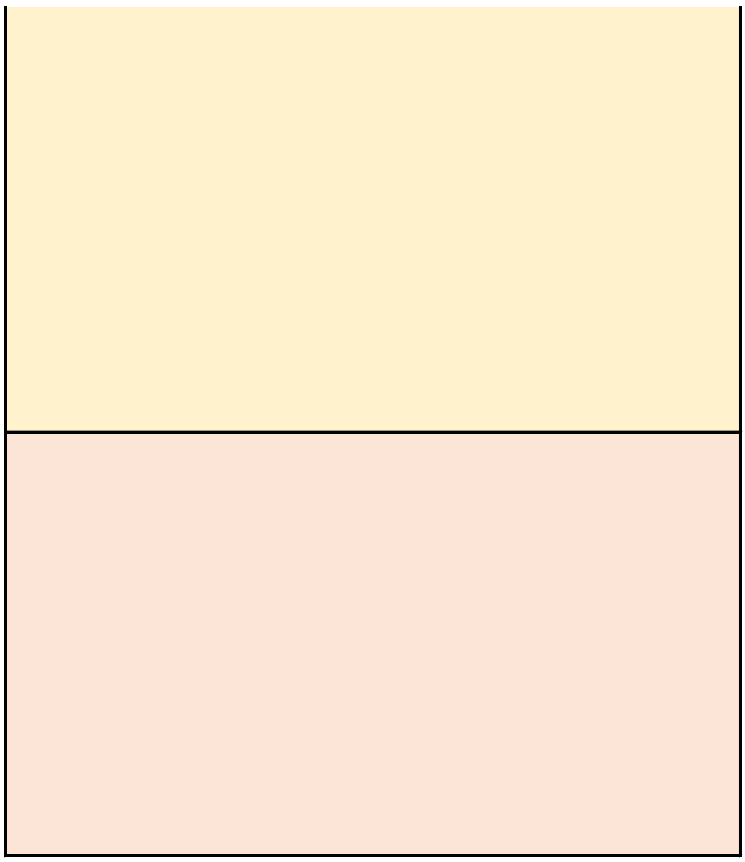

In the interest of transparency, we ask you to disclose all relationships/activities **consultation process**. "Related" means any relation with for-profit or not-for-pr in this activity. Disclosure represents a commitment to transparency and does n relationship/activity/interest, it is preferable that you do so.

		**Name all entities with whom you have this relationship or indicate none.**
		**Time frame: past 2 ye**
**1**	**Any financial support related to the current consultation process.**	
**3**	**Royalties or licenses**	
**4**	**Consulting fees**	
**5**	**Payment or honoraria for lectures, presentations, speakers bureaus, manuscript writing or educational events**	
**6**	**Support for attending meetings and/or travel**	
**7**	**Patents planned, issued or pending**	
**8**	**Stock or stock options**	
**9**	**Other financial or non-financial interests**	

s/interests listed below that are **related to the content of this**
rofit third parties whose interests may be affected by your participation
ıot necessarily indicate a bias. If you are in doubt about whether to list a

Specifications/Comments
ears

Results of the appropriateness exercise

N°	Intervention Area	Action
1	Diagnosis	Reinforce
2	Diagnosis	Include
3	Diagnosis	Modify
4	Diagnosis	Modify
5	Diagnosis	Modify

6	Diagnosis	Reinforce
7	Diagnosis	Include
8	Diagnosis	Include
9	Risk assessment	Include
10	Risk assessment	Modify
11	Risk assessment	Modify
12	Risk assessment	Include

**13**	**Risk assessment**	**Modify**
**14**	**Risk assessment**	**Include**
**15**	**Risk assessment**	**Modify**
**16**	**Risk assessment**	**Include**
**17**	**Risk assessment**	**Include**
**18**	**Risk assessment**	**Include**

19	Risk assessment	Include
20	Risk assessment	Include
21	Non-Pharmacologic treatment	Include
22	Non-Pharmacologic treatment	Include
23	Non-Pharmacologic treatment	Include
24	Non-Pharmacologic treatment	Include
25	Non-Pharmacologic treatment	Include
26	Non-Pharmacologic treatment	Include

**27**	**Non-Pharmacologic treatment**	**Include**
**28**	**Pharmacologic treatment**	**Reinforce**
**29**	**Pharmacologic treatment**	**Include**
**30**	**Pharmacologic treatment**	**Modify**
**31**	**Pharmacologic treatment**	**Include**
**32**	**Pharmacologic treatment**	**Modify**
**33**	**Pharmacologic treatment**	**Modify**
**34**	**Pharmacologic treatment**	**Modify**

**35**	**Pharmacologic treatment**	**Include**
**36**	**Pharmacologic treatment**	**Include**
**37**	**Pharmacologic treatment**	**Include**
**38**	**Pharmacologic treatment**	**Include**
**39**	**Pharmacologic treatment**	**Modify**
**40**	**Pharmacologic treatment**	**Modify**
**41**	**Continuity of care**	**Include**
**42**	**Continuity of care**	**Include**

43	Continuity of care	Include
44	Continuity of care	Include
45	Continuity of care	Include
46	Continuity of care	Include
47	Continuity of care	Modify
48	Continuity of care	Modify
49	Continuity of care	Include
50	Delivery System	Include

51	Delivery System	Include
52	Delivery System	Include
53	Vaccines	Modify
54	Vaccines	Modify
55	Vaccines	Modify
56	System for Monitoring	Include
57	System for Monitoring	Include

Improvement Proposal	Level of appropriateness for inc practice	
	Median of responses	Response rate
Exclusive use of clinically validated Blood Pressure Measuring Devices to accurate BP measurement	9	100%
A recommendation for BP mesurement in patients with atrial fibrillation.	6	97.4%
Simplify HTN diagnosis by using the 2nd BP measurement only when the 1st one is at least 130/80 mmHg.	5	100%
Simplify HTN diagnosis by using the 2nd BP measurement only when the 1st one is at least 140/90 mmHg. A 3rd measurement will be used if the difference between either the first two systolic or diastolic BPs was >5 mmHg.	5	100%
Avoid the 5 minutes of rest before BP measurement.	5	97.4%

Recommendations to improve BP measurement (unobserved, quiet room, etc).	7	100%
Expand HTN screening in the community served by each PHC (community engagement).	7.5	100%
BP thresholds to consider HTN in the step A (BP 140/90 in general population and SBP 130 in high CVD risk).	8	97.4%
Screening of CKD by urine albumin-creatinine ratio (uACR) and estimated Glomerular Filtration Rate (eGFR).	8	100%
De-prioritizing the use of CVD risk charts in CVD risk assessment.	5	92.1%
Clarify the CKD definition as follows: eGFR < 60 ml/min and/or AlbU/CrU index ≥ 30 mg/g.	8	97.4%
A case finding strategy (opportunistic screening) for Atrial Fibrillation, in high CVD risk patients of any age and in those ≥ 65 years, using a stepwise approach:	7.5	100%

radial pulse palpation to all and ECG in those with 1st test possitive.		
BP goals in elderly patients to SBP <130 (age ≥ 65 years as a high CVD-risk equivalent).	7.5	100%
History of HTN during pregnancy in the CVD risk assessment.	7	97.4%
Clarify the CVD risk approach for young adults (18 - 40 years) who are not covered by the CVD risk charts.	7	97.4%
A Recommendation to measure HTN-mediated organ damage with ECG in high CVD risk patients.	7	94.7%
Measure the heart rate variability using a smartphone (photopletysmography)	5	100%
Assess physcological distress using a simple tool such as Patient Health Questionnaire -2 Depression Screen.	6	100%

Screening for dyslipidimia and diabetes ammong patients with hypertension and obesity.	8	97.4%
Warning on the treatment of asymptomatic severe HTN to avoid referral to emergency department and acute treatment with short-acting/parenterally agents.	7	92.1%
A recommendation on the consumption of low-sodium / high-potassium salt.	8	97.4%
A recommendation on isometric exercise.	7	97.4%
A recommendation on sleep quality - Circadian Synchrony.	6	97.4%
Warning against Cannabis use.	7	97.4%
Warning against Electronic Cigarette use.	7	94.7%
A recommendation to avoid the sedentary lifestyle.	8	97.4%

A recommendation on excersice prescription.	8	97.4%
Use of fixed-dose combination.	9	100%
Recommendation of Triple FDC for those patients who don´t reach BP control using Double FDC.	7	94.7%
Add the third drug, at half maximum dose, in the second step of the treatment protocol instead increasing the first two drugs to maximum doses.	7	94.7%
Spironolactone in patients with 3 drugs at maximum doses and lack of HTN control.	7	97.4%
Statin dose in secondary prevention to Atorvastatin 80 mg or Rosuvastatin 40 mg.	8	89.5%
Statin dose in primary prevention to Atorvastatin 40 mg or Rosuvastatin 20 mg.	8	84.2%
Replace current medications in the treatment protocol with polypills (antihypertensive + statin +/- aspirin) for primary and secondary prevention.	7	89.5%

A recommendation for tobacco cessation treatment (e.g. bupropion, varenicline, nicotine substitutes)	7	92.1%
Recommendation to use iSGLT2 in patients with CKD.	8	92.1%
Recommendation to use iSGLT2 in patients with heart failure, regardless of ejection fraction.	8	89.5%
Recommendation to use iSGLT2 in patients with diabetes and established CVD.	8	89.5%
Reduce the intervals between steps for medication intensification to 2 weeks instead of 1 month.	7	97.4%
Change the warning "WOMEN of CHILDBEARING AGE" to "WOMEN of CHILDBEARING POTENTIAL"	6.5	94.7%
Recommendation on Home BP measurement for treatment monitoring.	8.5	100%
A target time to achieve BP control.	7	97.4%

Recommendation of using Telemedicine / mHealth apps to monitor adherence and compliance with recommendations, and to reduce absenteeism.	8	97.4%
Recommendation of assessing cognitive function to improve adherence.	6	97.4%
Lipid targets in high CVD risk patients.	8	94.7%
An advice not to discontinue statin therapy once the control target has been reached.	8	94.7%
Clarify that intensive BP goals only apply to patients <80 years**.**	7	97.4%
BP targets in high CVD risk patients should be BP < 130/80 rather SBP <130.	5	76.3%
A contact for patient support groups.	6	97.4%
Non-physician workers under supervision must follow patients and titrate medication to improve BP control and reduce CVD and mortality.	7	97.4%

Non-physician workers must perform HTN screening and CVD risk stratification.	7	97.4%
Non-physician workers must provide counseling on healthy life-style and medication adherence.	7	94.7%
Influenza vaccination to all patients with HTN, even those at low and moderate CVD risk.	7	97.4%
Indication for pneumococcus vaccination should exclude patients in primary prevention <65 years.	7	89.5%
Extend the vaccination to others conditions such as herpes, diphtheria-tetanus or hepatitis**.**	6	89.5%
A message about the importance of registering clinical variables.	8	97.4%
A message about the relevance of having a strategy of preformance evaluation with feedback.	8	97.4

corporating the	Highest tercile?
Response rate-weighted median	
9	YES
5.8	NO
5	NO
5	NO
4.9	NO

7	YES
7.5	YES
7.8	YES
8	YES
4.6	NO
7.8	YES
7.5	YES

7.5	YES
6.8	YES
6.8	YES
6.6	YES
5	NO
6	NO

7.8	YES
6.4	YES
7.8	YES
6.8	YES
5.8	NO
6.8	YES
6.6	YES
7.8	YES

7.8	YES
9	YES
6.6	YES
6.6	YES
6.8	YES
7.2	YES
6.7	YES
6.3	YES

6.4	YES
7.4	YES
7.2	YES
7.2	YES
6.8	YES
6.2	YES
8.5	YES
6.8	YES

7.8	YES
5.8	NO
7.6	YES
7.6	YES
6.8	YES
3.8	NO
5.8	NO
6.8	YES

6.8	YES
6.6	YES
6.8	YES
6.3	YES
5.4	NO
7.8	YES
7.8	YES

Agreed Interventions and their supports

Intervention Areas	Improvement proposals	Observational studies
**Diagnosis**	Exclusive use of clinically validated BP Measuring Devices to accurate BP measurement.	
	Improving BP measurement conditions.	X
	Expanding HTN screening in the community.	
	BP thresholds to consider HTN equal to 140/90 in general population and SBP 130 in high CVD risk patients.	X
**Risk assessment**	CKD screening by using uACR and eGFR.	X
	HTN-mediated organ damage assessment with ECG in high CVD risk patients.	X
	Dyslipidemia and diabetes screening among patients with HTN and obesity.	X
	CKD definition as follows: eGFR < 60 ml/min and/or AlbU/CrU index ≥ 30 mg/g.	X
	Opportunistic screening for AF in high CVD risk patients of any age and in those ≥ 65 years.	X
	Set BP goals in elderly patients to SBP <130 (age ≥ 65 years as a high CVD-risk equivalent).	X
	History of HTN during pregnancy for CVD risk assessment.	X
	CVD risk approach for young adults (18 - 40 years) who are not covered by the CVD risk charts.	X
	Warning on avoiding treatment of asymptomatic severe HTN with short-acting/parenterally agents.	X
**Non-Pharamacologic Treatment**	Use of low-sodium / high-potassium salt.	X
	Isometric exercise.	X
	Warning against smoking Cannabis.	X

**Treatment**	Warning against Electronic Cigarette use / Vaping.	X
	Avoid sedentary lifestyle.	X
	Exercise prescription.	X
**Pharmacologic Treatment**	FDC / single pill antihypertensives.	
	Triple FDC for those patients who don´t reach BP control using Double FDC.	
	Add the third drug, at half maximum dose, in the second step of the treatment protocol instead increasing the first two drugs to maximum doses.	
	Spironolactone in patients with 3 drugs at maximum doses and lack of HTN control.	
	Maximum statin dose in secondary prevention (Atorvastatin 80 mg or Rosuvastatin 40 mg).	
	Moderate statin dose in primary prevention (Atorvastatin 40 mg or Rosuvastatin 20 mg).	
	Use of Polypills for primary and secondary prevention.	
	Treatment for tobacco cessation (bupropion, varenicline, nicotine substitutes).	X
	Use of iSGLT2 in patients with CKD.	X
	Use of iSGLT2 in patients with heart failure.	X
	Use of iSGLT2 in patients with diabetes and established CVD.	
	Antihypertensive medication intensification intervals of 2 weeks.	X
	Warning on assessing childbeaing potential before treatment initiation.	X
	Home BP measurement for treatment monitoring.	X

**Continuity of Care**	Telemedicine / mHealth apps to monitor recommendation adherence and to reduce absenteeism.	X
	Lipid targets in high CVD risk patients	
	A established target time to achieve BP control.	X
	Warning to avoid statin discontinuation once the control target has been reached.	X
	Intensive BP goals restricted to patients <80 years.	X
**Delivery System**	Medication intensification by non-physician healthcare workers following a protocol.	X
	HTN screening and CVD risk stratification by non-physician healthcare workers.	X
	Healthy life-style counseling and medication adherence by non-physician helathcare workers .	X
**Vaccines**	Influenza vaccination to all patients with HTN regardless of CVD risk level.	
	Pneumococcus vaccination should exclude patients in primary prevention <65 years.	
**System for Monitoring**	Importance of registering clinical variables.	X
	Relevance of having a strategy of preformance evaluation with feedback.	X

AHA: American Hearts Association; ACC: American College of cardiology; ESC: European So
Hypertension; WHL: World Hypertension League; WHO: World Health Organization

HTN: hypertension; SBP: systolic blood pressure; uACR: urine albumin-creatinine ratio; eGF

Supporting Evidence			
**Randomized Clinical Trials**	**Systematic Review & Meta-analysis**	**Expert Consensus**	**AHA/ACC**
		X	X
		X	X
		X	X
X	X		
	X	X	X
	X	X	X
		X	X
X	X	X	X
		X	X
X	X		X
		X	
		X	X
		X	X
X	X		X
X	X		X
			X

		X	X
X	X		X
	X	X	X
X	X		X
X			
X			
X	X		X
X	X		X
X	X		X
X	X		
X	X		X
X			X
X	X		X
X	X		X
		X	
X	X		X

X	X	X	X
X	X	X	
		X	
	X	X	X
X			
X			X

ciety of Cardiology; ESH: European Society of Hypertension; LASH: Latin American Society of Hypertension;

FR: estimated glomerular filtration rate; ECG: electrocardiogram; AF: atrial fibrillation

Supporting Organizations						
**ESC/ESH**	**LASH**	**KDIGO**	**WHF**	**ISH**	**WHL**	**WHO**
X	X	X	X	X	X	X
X	X	X	X	X	X	X
X	X	X	X	X	X	
X						X
X	X	X		X		X
X	X	X	X	X		X
X	X	X	X	X		X
X	X	X		X		
X	X		X	X		X
X						
X						
X						
X				X		
X			X	X	X	X
X	X		X	X		
X						X

X					X	X
X	X	X	X	X	X	X
X	X	X	X	X	X	X
X	X		X	X	X	X
	X					
X						
X			X	X		
			X			
X			X			
X	X		X	X		X
X		X				X
X						X
X			X			X
X						X
X						
X	X		X	X		

X	X		X	X		X
X			X	X		
						X
X						
			X			X
X			X			
X			X			
			X			
			X			
			X			
			X			X

KDIGO: Kidney International; WHF: World Heart Federation; ISH: international Society of
